# Kinetic modelling reveals the presence of multistability in normal and stressful conditions in translational initiation mechanism

**DOI:** 10.1371/journal.pone.0319280

**Published:** 2025-03-21

**Authors:** Guturu L. Harika, Krishnamachari Sriram

**Affiliations:** 1 Center for Computational Biology, Department of Computational Biology, IIIT-Delhi, New Delhi, India; Pázmány Péter Catholic University: Pazmany Peter Katolikus Egyetem, HUNGARY

## Abstract

Protein synthesis involves translation initiation, elongation, termination, and ribosome recycling, and each step is controlled intricately by many signaling proteins. Translation initiation can be compactly categorized into two mechanisms: primary and secondary. The primary mechanism involves the recruitment of three important eukaryotic initiation factors, eIF2-GDP, eIF5, and eIF2B, and their interactions, followed by the GDP-GTP exchange by eIF2B to form an active dimer eIF2-GTP. The dimer binds with Met-tRNA to form a robust ternary complex (TC). The secondary mechanism closely mirrors the primary reaction mechanism, except that the interactions of eIF2B and eIF5 happen with the TC to form complexes. These interactions happen with high fidelity and precision, failing which fail-safe mechanisms are invoked instantaneously to delay the initiation process. In this work, we build a mathematical model to unravel how the transition between translation initiation and termination occurs at the initiation stage based on the elementary mechanisms we built from the network assembled from experimental observations. We focus only on the dynamics of primary and secondary mechanisms involved in the translation initiation process under normal and integrated stress response (ISR) conditions that act as a fail-safe mechanism by through phosphorylation-dephosphorylation (PdP) reactions. Since the network is huge and has many unknown kinetic parameters, we perform structural analysis using chemical reaction network theory (CRNT) and find hidden positive feedback loops that regulate the initiation mechanism. We apply bifurcation theory to show that the model exhibits ultrasensitivity and bistability under normal conditions, while under ISR, it exhibits both bistability and tristability for the choice of kinetic parameters. We attribute bistability to translation initiation and termination and tristability in ISR to translation recovery and attenuation. We conclude that the translation initiation process is a highly regulated process guided by the threshold and switching mechanisms to make quick decisions on the translation initiation, termination, recovery or attenuation under different conditions.

## Introduction

In eukaryotic cells, translation involves initiation, elongation, termination, and ribosome recycling. Translation initiation is a complex process involving many eukaryotic initiation factors (eIFs) proteins, and presently, there are as many as 12 eukaryotic initiation factors known to play a vital role. Among these initiation factors, eIF2 (e2), eIF2B (e2B), and eIF5’s (e5) roles have been thoroughly elucidated and have helped us understand the inner workings of regulating protein synthesis [1].

Two mechanisms operate in the translation initiation process: primary and secondary. The role of initiation factors e5 and e2B and their interactions with eIF2-GDP (e2GDP) is well documented [2]. The initiation factor e5 acts as a GDP dissociation inhibitor (GDI) in the primary mechanism and GTPase activating protein (GAP) in the secondary mechanism. The initiation factor e2B acts as a Guanine nucleotide exchange factor (GEF) in the primary mechanism and the GDI displacement factor (GDF) in the primary and secondary mechanisms [3,4]. The primary mechanism in the translation initiation process begins with the e2, which binds to GTP and Met-tRNA (tR) to form a ternary complex (TC) [5]. e2 consists of three protein subunits αβγ. The formation of TC is a multistep process involving recruiting two other initiation factors, e2B and e5. e2B facilitates GDP-GTP exchange of e2 and enables the binding of tR to form TC. Though the role of e2B is known, the exact step-wise mechanisms of this process are unknown. e2B is a multi-complex consisting of five protein subunits αβγδε [6]. These subunits are vital, and individual subunits have a unique role in translation initiation. e5 has two activities: it is a GAP for the eIF2-GTP (e2GTP) binary complex and a GDI that attenuates translation.

The secondary mechanism involves again the initiation factors e5 and e2B interactions with TC. These reactions mirror the primary mechanism, but in the end, the initiation factors dissociate TC to form e2GDP that recycles for the next round of the initiation process. Eukaryotic factors e5 and e2B are vital in cycling e2 between active and inactive states. These three eukaryotic factors and GTP form a regulatory circuit to control the initiation of the translational process. The eukaryotic factor e5 accelerates the translation process after the formation of TC under normal conditions, whereas e2B slows the process under unfavourable conditions [7]. Under normal conditions, the recycling of active e2 happens with e5 binding to the complex and activating the GTP hydrolysis. The resulting complex eIF2-GDP-eIF5 (Ce1) is inactive. Following this, e2B binds to this complex, displaces e5, and exchanges GDP to GTP to form the complex e2GTP to allow the tR binding to form TC.

However, translation initiation mechanisms are complex, and a static signalling network alone cannot completely help to understand its behaviour under normal conditions. In this work, we convert this static network to a set of elementary chemical steps and, further, convert it into sets of nonlinear differential equations to study its solution using the tools of bifurcation theory. We present an ordinary differential equation (ODE) model for the translation initiation mechanism, including both primary and secondary mechanisms, and address their role under normal conditions. We show that the network has hidden positive feedback due to the complex interactions involving e2B and e5 with the e2GDP and TC. The model exhibits ultrasensitivity, biphasic, and bistability, when choosing appropriate parameters. We relate these dynamics to molecular controls in the translation process and provide insight into how the hidden positive feedback provides a threshold to signal the initiation and termination of the translation process. We also extend our study to the behaviour of translation initiation under stressful conditions in the ISR, where we model the dynamics of PdP reactions that attenuate the initiation process and show that the model exhibits tristability. We map and explain the occurrence of tristable dynamics to the recovery and attenuation process.

We start by writing all the important chemical steps in detail based on the network we construct only from the experimental observations. We also analyse the network based on the chemical reaction network theory to determine the presence of a positive feedback loop in the network by determining the deficiency of the network. Finally, we apply the dynamical systems theory to show that the kinetic modelling of normal and stressful events exhibits multistability, and we explain and discuss the implications of these dynamics.

## Translation initiation network under normal conditions, their biochemical steps, and ODEs

We base all the reactions below on the laws of mass action kinetics. We map the biochemical steps directly from the translation initiation network under normal conditions. The rate constants kf_i_’s and kb_i_’s are i^th^ forward and backward reactions, respectively. Ri’s are the reactions of the i^th^ biochemical step, and R_i_’s are the corresponding reaction rates.

The network under normal conditions comprises 13 reactions. These reactions can be divided into three modules: Primary mechanism (PM), Secondary mechanism (SM), and GDP-GTP exchange/Met-tRNA reactions. In this section, we explain all the reactions and give each module’s corresponding set of reactions.

### Module-1 (M1): Primary mechanism (PM)

**Fig 1:**
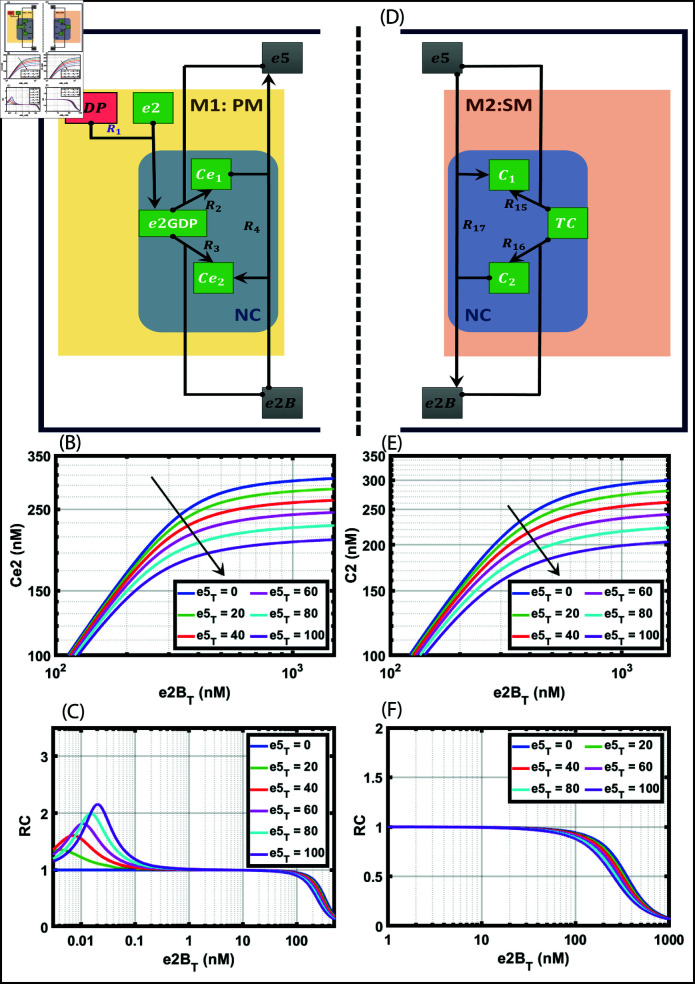
Module 1 (M1), primary mechanism (PM), and module 2 (M2), secondary mechanism (SM) under normal conditions (NC). A: Network of e5 and e2B binding with e2GDP along with the competition and displacement reaction in PM. Along with the competition between e5 and e2B, we introduce an additional displacement reaction in which e2B displaces e5 from e2GDP. B: Bifurcation diagram of Ce_2_ with total e2B (e2B_T_) as the parameter with different concentrations of total e5 (e5_T_) ranging from 0 – 100 nM. C: The response coefficient (RC) plot of Ce_2_ with e2B_T_ as the parameter. D: Network of e5 and e2B binding with TC along with the competition and displacement reaction in SM. Along with the competition between e5 and e2B, e5 displaces e2B from TC. E: Bifurcation diagram of C_2_ with total e2B (e2B_T_) as the parameter with different concentrations of total e5 (e5_T_) ranging from 0 – 100 nM. F: The response coefficient (RC) plot of C_2_ with e2B_T_ as the parameter. The RC is given by $d(ln(Ce_{2}))∕d(ln(e2B_{T}))$. The XPPAUT files used for simulations is in the supplementary S7 Fig1A.ode, and S8 Fig1D.ode.

**Fig 2:**
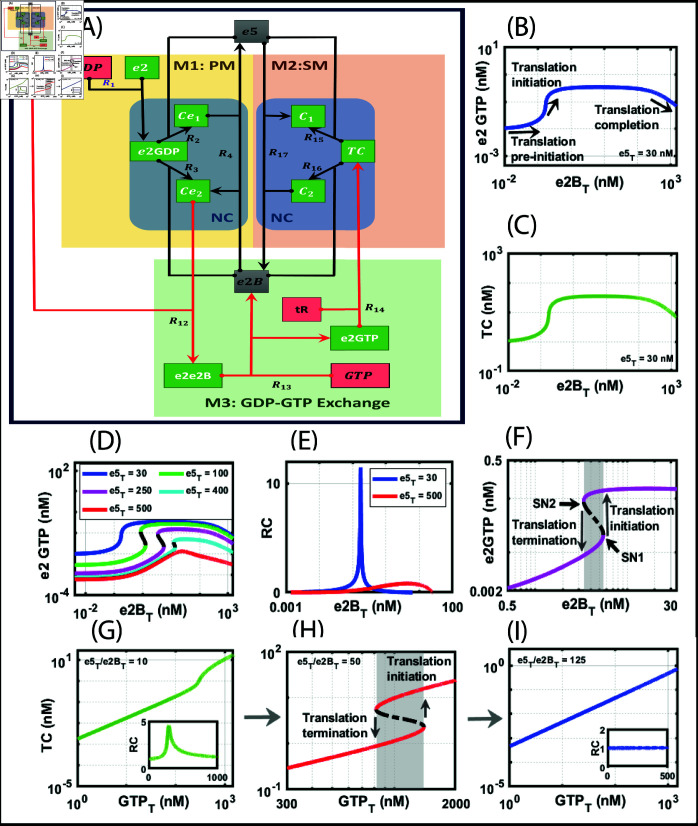
Module 1 (M1), primary mechanism (PM) and Module 2 (M2), secondary mechanism (SM), and Module 3 (M3) GDP-GTP exchange under normal conditions (NC). A: Network of translation initiation under normal conditions. B: Bifurcation diagram of e2GTP with e2B_T_ as parameter keeping e5_T_ at 30 nM showing ultrasensitive and biphasic dynamics. C: Bifurcation diagram of TC with e2B_T_ as parameter keeping e5_T_ at 30 nM showing ultrasensitive and biphasic dynamics. D: Bifurcation diagram of e2GTP with total e2B (e2B_T_) as the parameter with different concentrations of total e5 (e5_T_) ranging from 30 – 500 nM. E: The response coefficient (RC) plot of e2GTP with e2B_T_ as the parameter. The RC is given by $d(ln(e2GTP))∕d(ln(e2B_{T}))$. The RC is shown only for the ultrasensitive plots. F: Bifurcation of e2GTP with e2B_T_ as the parameter showing bistable dynamics. With the increase in e2B_T_ concentration, the system switches from a lower steady state to a higher steady state, corresponding to termination and initiation of translation, respectively. The total concentrations of free e2, e5, tR, GTP, and GDP were kept constant at 500, 250, 2500, 1200 and 40 nM, respectively. G: Bifurcation diagram of TC with total GTP (GTP_T_) as parameter keeping the ratio of $\frac{\text{e}5_{\text{T}}}{\text{e}2{\text{B}}_{\text{T}}}=10$ showing ultrasensitive dynamics. The RC plot in the inset shows ultrasensitivity. H: Bifurcation diagram of TC with GTP_T_ as parameter keeping the ratio of $\frac{\text{e}5_{\text{T}}}{\text{e}2{\text{B}}_{\text{T}}}=50$ showing bistable dynamics. I: Bifurcation diagram of TC with GTP_T_ as parameter keeping the ratio of $\frac{\text{e}5_{\text{T}}}{\text{e}2{\text{B}}_{\text{T}}}=125$ showing graded response. The inset’s RC plot shows a graded response. The XPPAUT file used for simulations is in the Supporting information S9 Fig2A.ode.

We show the M1 of the network in Fig 1A and Fig 2A left. We start with the interactions of three important initiation factors: e2GDP, e5, and e2B. The reactions R1 and R2 below capture the competition between e5 and e2B to bind with e2GDP, respectively.

In the reaction R1, e2 binds with GDP to form e2GDP [2]. The e2GDP further interacts with e5 and e2B for the translation initiation.


$$\begin{array}{llllllllllll} & \text{R1}:\; & \text{e2}+\text{GDP} & \overset{{\text{kf}}_{1}}{\underset{{\text{kb}}_{1}}{⇌}}\text{e2GDP}\; & {\text{R}}_{1} & ={\text{kf}}_{1}[\text{e2}][\text{GDP}]-{\text{kb}}_{1}[\text{e2GDP}]\; & & \; & & \; & & \; \end{array}$$

In reaction R2, e5 binds with the β subunit of e2GDP to form a complex Ce_1_. Since e5 is a GDI, the binding of e5 to e2GDP prevents GDP release from e2 [2]. This binding helps in translation control by preventing GDP release without e2B.


$$\begin{array}{llllllllllll} & \text{R2}:\; & \text{e2GDP}+\text{e5} & \overset{{\text{kf}}_{2}}{\underset{{\text{kb}}_{2}}{⇌}}{\text{Ce}}_{1}\; & {\text{R}}_{2} & ={\text{kf}}_{2}[\text{e2GDP}][\text{e5}]-{\text{kb}}_{2}[{\text{Ce}}_{1}]\; & & \; & & \; & & \;\\ & \text{R3}:\; & \text{e2GDP}+\text{e2B} & \overset{{\text{kf}}_{3}}{\underset{{\text{kb}}_{3}}{⇌}}{\text{Ce}}_{2}\; & {\text{R}}_{3} & ={\text{kf}}_{3}[\text{e2GDP}][\text{e2B}]-{\text{kb}}_{3}[{\text{Ce}}_{2}]\; & & \; & & \; & & \; \end{array}$$

The e2B regulatory site has three subunits: α, β, and δ. The e2B also has a catalytic site with two subunits, namely γ and ε, with the γ subunit binding to and stimulating the GEF action of the ε subunit of e2B [3,8]. In reaction R3, we consider the ε subunit of e2B interacting with the β subunit of e2, while the GDP bound to the γ subunit of e2, forming a complex Ce_2_.

The catalytic site of e2B is known to actively displace e5 from Ce_1_ [2]. The reaction, R4 below, shows the displacement reaction of e5 by e2B from e2GDP. This displacement reaction gives the complex Ce_2_.


$$\begin{array}{llllllllllll} & \text{R4}:\; & {\text{Ce}}_{1}+\text{e2B} & \overset{{\text{kf}}_{4}}{\underset{{\text{kb}}_{4}}{⇌}}{\text{Ce}}_{2}+\text{e5}\; & {\text{R}}_{4} & ={\text{kf}}_{4}[{\text{Ce}}_{1}][\text{e2B}]-{\text{kb}}_{4}[{\text{Ce}}_{2}][\text{e5}]\; & & \; & & \; & & \; \end{array}$$

### Module-2 (M2): Secondary mechanism (SM)

We show the network for M2 in Fig 1D, and Fig 2A right that closely mirrors M1. The competition of e5 and e2B for e2 exists even after the formation of the TC [2]. After the formation of TC (We show this as reaction R14 in Module-3 below), in the reactions R15 and R16 below, we show the competition between e5 and e2B for TC. The TC binds with e5 and e2B to form the complexes C_1_ and C_2_, respectively.


$$\begin{array}{llllllllllll} & \text{R15}:\; & \text{TC}+\text{e5} & \overset{{\text{kf}}_{15}}{\underset{{\text{kb}}_{15}}{⇌}}{\text{C}}_{1}\; & {\text{R}}_{15} & ={\text{kf}}_{15}[\text{TC}][\text{e5}]-{\text{kb}}_{15}[{\text{C}}_{1}]\; & & \; & & \; & & \;\\ & \text{R16}:\; & \text{TC}+\text{e2B} & \overset{{\text{kf}}_{16}}{\underset{{\text{kb}}_{16}}{⇌}}{\text{C}}_{2}\; & {\text{R}}_{16} & ={\text{kf}}_{16}[\text{TC}][\text{e2B}]-{\text{kb}}_{16}[{\text{C}}_{2}]\; & & \; & & \; & & \; \end{array}$$

TC forms a stable complex with e5 [2]. Hence, we added reactions to include the displacement of e2B from TC by e5 (reaction R17 below). In reaction R17, we show the displacement of e2B by e5 to form the complex C_1_


$$\begin{array}{llllllllllll} & \text{R17}:\; & {\text{C}}_{2}+\text{e5} & \overset{{\text{kf}}_{17}}{\underset{{\text{kb}}_{17}}{⇌}}{\text{C}}_{1}+\text{e2B}\; & {\text{R}}_{17} & ={\text{kf}}_{17}[{\text{C}}_{2}][\text{e5}]-{\text{kb}}_{17}[{\text{C}}_{1}][\text{e2B}]\; & & \; & & \; & & \; \end{array}$$

### Module-3 (M3): Coupling of PM and SM by GDP-GTP exchange

The reactions R12 - R14 below represent M3. In the reaction R12 below, the complex Ce_2_ releases GDP, leaving behind the e2e2B complex. In the reaction R13, e2B mediates the exchange of GDP to GTP, and in the reaction R14, tR binds with e2GTP to form TC.


$$\begin{array}{llllllllllll} & \text{R12}:\; & {\text{Ce}}_{2} & \overset{{\text{kf}}_{12}}{\underset{{\text{kb}}_{12}}{⇌}}\text{e2e2B}+\text{GDP}\; & {\text{R}}_{12} & ={\text{kf}}_{12}[{\text{Ce}}_{2}]-{\text{kb}}_{12}[\text{e2e2B}][\text{GDP}]\; & & \; & & \; & & \;\\ & \text{R13}:\; & \text{e2e2B}+\text{GTP} & \overset{{\text{kf}}_{13}}{\underset{{\text{kb}}_{13}}{⇌}}\text{e2GTP}+\text{e2B}\; & {\text{R}}_{13} & ={\text{kf}}_{13}[\text{e2e2B}][\text{GTP}]-{\text{kb}}_{13}[\text{e2GTP}][\text{e2B}]\; & & \; & & \; & & \;\\ & \text{R14}:\; & \text{tR}+\text{e2GTP} & \overset{{\text{kf}}_{14}}{\underset{{\text{kb}}_{14}}{⇌}}\text{TC}\; & {\text{R}}_{14} & ={\text{kf}}_{14}[\text{tR}][\text{e2GTP}]-{\text{kb}}_{14}[\text{TC}]\; & & \; & & \; & & \; \end{array}$$

## Structural analysis of the network using CRNT

We carry out the structural analysis of the network using CRNT for all the three modules (M1-M3) together. We use the CRNToolbox (https://cbe.osu.edu/chemical-reaction-network-theory) to get information about the network’s structural aspects based on the network’s deficiency. Importantly, we find whether the network can exhibit multistability without knowing the kinetic parameters. CRNT also provides information about the number of complexes, linkage classes, rank, and the deficiency of the entire network. Based on the number of complexes (c), linkage classes (l), and the rank of the network (r), the deficiency (δ) of the network is given in the Eq 1.


$$\begin{array}{c} \delta =\text{c}-\text{l}-\text{r} \end{array}$$ (1)

From the CRNToolbox, we determine the deficiency and the presence of a positive feedback loop in the network. The details are provided in the respective sections along with ODEs. All the .NET files used in this work are provided in the Supporting information S1 Fig1A.NET–S6 Fig4.NET.

## Sensitivity analysis

To quantify ultrasensitivity, we calculate and plot the response coefficient (RC) or logarithmic gain using the following expression [9].


RC= lim ⁡ δM→0 (MTM)δMδMT=d[log[M]]d[log[MT]]

The RC in the above equation is the derivative of the **transfer function**, which models the change in the system output (M) to its corresponding input (M_T_). The model exhibits ultrasensitivity when RC > 1 and exhibits a steep rise, reaches the peak, and then falls slowly in the logarithmic coordinates. The peak is the maximum value of RC > 1, which indicates ultrasensitivity.

## Normal conditions: Only ultrasensitivity results in PM due to competition and displacement

Since the network is huge and the mechanisms are complex, we start with the numerical analysis of PM to understand the behaviour of the translation process. We mark the four important reactions in the network (Fig 1A): R1–R4, which captures the formation of e2GDP and the competition between e5 and e2B for e2GDP that forms complexes Ce_1_ and Ce_2_, respectively. The e5 competition delays the e2B binding to e2GDP and the translation process, and e2B should displace the e5 from the complex Ce_1_ to form the complex Ce_2_. We believe competition and displacement may bring in threshold and ultrasensitivity dynamics. To verify, we perform a simulation to study the stand-alone effect of e5 and e2B to determine the type of dynamics the network can exhibit. The corresponding ODEs and the mass conservation relations are in Table 1 (row 1). We also want to check whether the network can exhibit bistable dynamics, and for that, we subject PM network to the CRNT analysis.

**Table 1:** Network summary.

S. No.	Networks	Module	Reactions	ODEs	Mass Conservation	CRNT	File	Other Dynamics	Biological significance
Normal Conditions									
1	Fig 1A	M1: PM-NC	R1-R4	$\frac{\text{d}[\text{e}5]}{\text{d}\text{t}}=-{\text{R}}_{2}+{\text{R}}_{4}$	${\text{e2B}}_{\text{T}}=[\text{e2B}]+[{\text{Ce}}_{2}]$	δ = 1	S7 Fig1A.ode	Ultrasensitivity,	PM-NC: Competition between e5 and e2B for e2
				$\frac{\text{d}[\text{e2B}]}{\text{dt}}=-{\text{R}}_{3}-{\text{R}}_{4}$	${\text{e5}}_{\text{T}}=[\text{e5}]+[{\text{Ce}}_{1}]$			Graded response	Displacement of e5 by e2B
				$\frac{\text{d}[{\text{Ce}}_{2}]}{\text{dt}}={\text{R}}_{3}+{\text{R}}_{4}$	${\text{GDP}}_{\text{T}}=[\text{GDP}]+[{\text{Ce}}_{1}]+[{\text{Ce}}_{2}]+[\text{e2GDP}]$				Acceleration: e2B
				$\frac{\text{d}[\text{e}2]}{\text{dt}}=-{\text{R}}_{1}$	${\text{e2}}_{\text{T}}=[\text{e2}]+[{\text{Ce}}_{1}]+[{\text{Ce}}_{2}]+[\text{e2GDP}]$				Brake: e5
				$\frac{\text{d}[\text{GDP}]}{\text{dt}}=-{\text{R}}_{1}$					
				$\frac{\text{d}[\text{e2GDP}]}{\text{dt}}={\text{R}}_{1}-{\text{R}}_{2}-{\text{R}}_{3}$					
				$\frac{\text{d}[{\text{Ce}}_{1}]}{\text{dt}}={\text{R}}_{2}-{\text{R}}_{4}$					
2	Fig 1D	M2: SM-NC	R15-R17	$\frac{\text{d}[\text{e5}]}{\text{dt}}=-{\text{R}}_{15}-{\text{R}}_{17}$	${\text{e2B}}_{\text{T}}=[\text{e2B}]+[\text{C2}]$	δ = 1	S8 Fig1D.ode	Ultrasensitivity,	SM-NC: Competition between e5 and e2B for e2
				$\frac{\text{d}[\text{e2B}]}{\text{dt}}=-{\text{R}}_{16}+{\text{R}}_{17}$	${\text{e5}}_{\text{T}}=[\text{e5}]+[\text{C1}]$			Graded response	Displacement of e2B by e5
				$\frac{\text{d}[\text{C2}]}{\text{dt}}={\text{R}}_{16}-{\text{R}}_{17}$	${\text{TC}}_{\text{T}}=[\text{TC}]+[\text{C1}]+[\text{C2}]$				Acceleration: e5
				$\frac{\text{d}[\text{TC}]}{\text{dt}}=-\text{R}15-{\text{R}}_{16}$					Brake: e2B
				$\frac{\text{d}[\text{C1}]}{\text{dt}}={\text{R}}_{15}+{\text{R}}_{17}$					
3	Fig 2A	M1 : PM-NC	R1-R4	$\frac{\text{d}[\text{e5}]}{\text{dt}}=-{\text{R}}_{2}+{\text{R}}_{4}-{\text{R}}_{15}-{\text{R}}_{17}$	${\text{tR}}_{\text{T}}=[\text{tR}]+[{\text{C}}_{1}]+[{\text{C}}_{2}]+[\text{TC}]$	δ = 2	S9 Fig2A.ode	Ultrasensitivity,	PM-NC: Competition between e5 and e2B for e2
		M2: SM-NC	R15-R17	$\frac{\text{d}[\text{e2B}]}{\text{dt}}=-{\text{R}}_{3}-{\text{R}}_{4}+{\text{R}}_{13}-{\text{R}}_{16}+{\text{R}}_{17}$	${\text{GTP}}_{\text{T}}=[\text{GTP}]+[{\text{C}}_{1}]+[{\text{C}}_{2}]+[\text{TC}]+[\text{e2GTP}]$	Bistability		Biphasic,	Displacement of e5 by e2B
		M3: GDP-GTP	R12-R14	$\frac{\text{d}[{\text{Ce}}_{2}]}{\text{dt}}={\text{R}}_{3}+{\text{R}}_{4}-{\text{R}}_{12}$	${\text{e2B}}_{\text{T}}=[\text{e2B}]+[{\text{C}}_{2}]+[{\text{Ce}}_{2}]+[\text{e2e2B}]$			Threshold	Acceleration: e2B
				$\frac{\text{d[TC]}}{\text{dt}}={\text{R}}_{14}-{\text{R}}_{15}-{\text{R}}_{16}$	${\text{e5}}_{\text{T}}=[\text{e5}]+[{\text{C}}_{1}]+[{\text{Ce}}_{1}]$				Brake: e5
				$\frac{\text{d[e2]}}{\text{dt}}=-{\text{R}}_{1}$	${\text{GDP}}_{\text{T}}=[\text{GDP}]+[{\text{Ce}}_{1}]+[{\text{Ce}}_{2}]+[\text{e2GDP}]$				
				$\frac{\text{d[GDP]}}{\text{dt}}={\text{R}}_{12}-{\text{R}}_{1}$	${\text{e2}}_{\text{T}}=[\text{e2}]+[{\text{C}}_{1}]+[{\text{C}}_{2}]+[{\text{Ce}}_{1}]+[{\text{Ce}}_{2}]$				SM-NC: Competition between e5 and e2B for e2
				$\frac{\text{d}[\text{e2GDP}]}{\text{dt}}={\text{R}}_{1}-{\text{R}}_{2}-{\text{R}}_{3}$	+[TC]+[e2GDP]+[e2GTP]+[e2e2B]				Displacement of e2B by e5
				$\frac{\text{d}[{\text{Ce}}_{1}]}{\text{dt}}={\text{R}}_{2}-{\text{R}}_{4}$					Acceleration: e5
				$\frac{\text{d}[{\text{C}}_{1}]}{\text{dt}}={\text{R}}_{15}+{\text{R}}_{17}$					Brake: e2B
				$\frac{\text{d}[{\text{C}}_{2}]}{\text{dt}}={\text{R}}_{16}-{\text{R}}_{17}$					
				$\frac{\text{d}[\text{GTP}]}{\text{dt}}=-\text{R}13$					
				$\frac{\text{d}[\text{tR}]}{\text{dt}}=-{\text{R}}_{14}$					
				$\frac{\text{d}[\text{e2e2B}]}{\text{dt}}=\text{R}12-\text{R}13$					
				$\frac{\text{d}[\text{e2GTP}]}{\text{dt}}=\text{R}13-\text{R}14$					
Integrated Stress Response									
4	Fig 3A	M1: PM	R1-R11	$\frac{\text{d[e5]}}{\text{dt}}=-{\text{R}}_{2}+{\text{R}}_{4}-{\text{R}}_{9}+{\text{R}}_{11}$	${\text{kin}}_{\text{T}}=[\text{kin}]+[\text{e2kin}]$	δ = 3	S10 Fig3A.ode	Ultrasensitivity,	PM-NC: Competition between e5 and e2B for e2
				$\frac{\text{d}[\text{e2B}]}{\text{dt}}=-{\text{R}}_{3}-{\text{R}}_{4}-{\text{R}}_{10}-{\text{R}}_{11}$	${\text{PP}}_{\text{T}}=[\text{PP}]+[\text{e2pPP}]$	Bistability		Threshold	Displacement of e5 by e2B
				$\frac{\text{d}[{\text{Ce}}_{2}]}{\text{dt}}={\text{R}}_{3}+{\text{R}}_{4}$	${\text{e2B}}_{\text{T}}=[\text{e2B}]+[{\text{Ce}}_{2}]+[{\text{Ce}}_{\text{4p}}]$				Acceleration: e2B
				$\frac{\text{d[kin]}}{\text{dt}}=-{\text{R}}_{5}+{\text{R}}_{6}$	${\text{e5}}_{\text{T}}=[\text{e5}]+[{\text{Ce}}_{1}]+[{\text{Ce}}_{\text{3p}}]$				Brake: e5
				$\frac{\text{d[PP]}}{\text{dt}}=-{\text{R}}_{7}+{\text{R}}_{8}$	${\text{GDP}}_{\text{T}}=[\text{GDP}]+[\text{e2GDP}]+[\text{e2pGDP}]+[\text{e2kin}]$				
				$\frac{\text{d[e2]}}{\text{dt}}=-{\text{R}}_{1}$	$+[\text{e2pPP}]+[{\text{Ce}}_{1}]+[{\text{Ce}}_{2}]+[{\text{Ce}}_{\text{3p}}]+[{\text{Ce}}_{\text{4p}}]$				PM-SC: Competition between e5 and e2B for e2
				$\frac{\text{d[GDP]}}{\text{dt}}=-{\text{R}}_{1}$	${\text{e2}}_{\text{T}}=[\text{e2}]+[{\text{Ce}}_{1}]+[{\text{Ce}}_{2}]+[\text{e2GDP}]+[{\text{Ce}}_{\text{3p}}]$				Displacement of e5 by e2B
				$\frac{\text{d[e2GDP]}}{\text{dt}}={\text{R}}_{1}-{\text{R}}_{2}-{\text{R}}_{3}-{\text{R}}_{5}+{\text{R}}_{8}$	$+[{\text{Ce}}_{\text{4p}}]+[\text{e2kin}]+[\text{e2pPP}]$				Acceleration: -
				$\frac{\text{d}[{\text{Ce}}_{1}]}{\text{dt}}={\text{R}}_{2}-{\text{R}}_{4}$					Brake: e2B, e5
				$\frac{\text{d[e2kin]}}{\text{dt}}={\text{R}}_{5}-{\text{R}}_{6}$					
				$\frac{\text{d[e2pPP]}}{\text{dt}}={\text{R}}_{7}-{\text{R}}_{8}$					
				$\frac{\text{d[e2pGDP]}}{\text{dt}}={\text{R}}_{6}-{\text{R}}_{7}-{\text{R}}_{9}-{\text{R}}_{10}$					
				$\frac{\text{d}[{\text{Ce}}_{4p}]}{\text{dt}}={\text{R}}_{10}+{\text{R}}_{11}$					
				$\frac{\text{d}[{\text{Ce}}_{3p}]}{\text{dt}}={\text{R}}_{9}-{\text{R}}_{11}$					
5	Fig 3D	M2: SM	R15-R24	$\frac{\text{d[e5]}}{\text{dt}}=-{\text{R}}_{15}-{\text{R}}_{17}-{\text{R}}_{22}+{\text{R}}_{24}$	${\text{kin}}_{\text{T}}=[\text{kin}]+[\text{TCkin}]$	δ = 3	S11 Fig3D.ode	Ultrasensitivity,	PM-NC: Competition between e5 and e2B for e2
				$\frac{\text{d}[\text{e2B}]}{\text{dt}}=-{\text{R}}_{16}+{\text{R}}_{17}-{\text{R}}_{23}-{\text{R}}_{24}$	${\text{PP}}_{\text{T}}=[\text{PP}]+[\text{TCpPP}]$	Bistability		Threshold	Displacement of e2B by e5
				$\frac{\text{d}[\text{C2}]}{\text{dt}}={\text{R}}_{16}-{\text{R}}_{17}$	${\text{e2B}}_{\text{T}}=[\text{e2B}]+[\text{C2}]+[\text{C4p}]$				Acceleration: e5
				$\frac{\text{d}[\text{kin}]}{\text{dt}}=-{\text{R}}_{18}+{\text{R}}_{19}$	${\text{e5}}_{\text{T}}=[\text{e5}]+[{\text{C}}_{1}]+[{\text{C}}_{\text{3p}}]$				Brake: e2B
				$\frac{\text{d}[\text{PP}]}{\text{dt}}=-{\text{R}}_{20}+{\text{R}}_{21}$	${\text{TC}}_{\text{T}}=[\text{TC}]+[{\text{C}}_{1}]+[{\text{C}}_{2}]+[{\text{C}}_{3p}]+[{\text{C}}_{\text{4p}}]$				
				$\frac{\text{d}[\text{TC}]}{\text{dt}}=-{\text{R}}_{15}-{\text{R}}_{16}-{\text{R}}_{18}+{\text{R}}_{21}$	$+[\text{TCkin}]+[{\text{TC}}_{\text{p}}]+[{\text{TC}}_{\text{pPP}}]$				SM-SC: Competition between e5 and e2B for e2
				$\frac{\text{d}[{\text{C}}_{1}]}{\text{dt}}={\text{R}}_{15}+{\text{R}}_{17}$					Displacement of e5 by e2B
				$\frac{\text{d}[\text{TCkin}]}{\text{dt}}={\text{R}}_{18}-{\text{R}}_{19}$					Acceleration: -
				$\frac{\text{d}[{\text{TC}}_{\text{pPP}}]}{\text{dt}}={\text{R}}_{20}-{\text{R}}_{21}$					Brake: e2B, e5
				$\frac{\text{d}[{\text{TC}}_{\text{p}}]}{\text{dt}}={\text{R}}_{19}-{\text{R}}_{20}-{\text{R}}_{22}-{\text{R}}_{23}$					
				$\frac{\text{d}[{\text{C}}_{\text{4p}}]}{\text{dt}}={\text{R}}_{23}+{\text{R}}_{24}$					
				$\frac{\text{d}[{\text{C}}_{\text{3p}}]}{\text{dt}}={\text{R}}_{22}-{\text{R}}_{24}$					
6	Fig 4	M1: PM	R1-R24	$\frac{\text{d[e5]}}{\text{dt}}=-{\text{R}}_{2}+{\text{R}}_{4}-{\text{R}}_{9}+{\text{R}}_{11}-{\text{R}}_{15}-{\text{R}}_{17}-{\text{R}}_{22}+{\text{R}}_{24}$	${\text{kin}}_{\text{T}}=[\text{kin}]+[\text{TCkin}]+[\text{e2kin}]$	δ = 6	S12 Fig4.ode	Bistability,	PM-NC: Competition between e5 and e2B for e2
		M2: SM		$\frac{\text{d[e2B]}}{\text{dt}}=-{\text{R}}_{3}-{\text{R}}_{4}-{\text{R}}_{10}-{\text{R}}_{11}+{\text{R}}_{13}-{\text{R}}_{16}+{\text{R}}_{17}-{\text{R}}_{23}-{\text{R}}_{24}$	${\text{tR}}_{\text{T}}=[\text{tR}]+[{\text{C}}_{1}]+[{\text{C}}_{2}]+[{\text{C}}_{\text{3p}}]$	Tristability		Ultrasensitivity,	Displacement of e2B by e5
		M3: GDP-GTP		$\frac{\text{d}[{\text{Ce}}_{2}]}{\text{dt}}={\text{R}}_{3}+{\text{R}}_{4}-{\text{R}}_{12}$	$+[{\text{C}}_{\text{4p}}]+[\text{TCkin}]+[{\text{TC}}_{\text{p}}]+[\text{TCpPP}]+[\text{TC}]$			Threshold	Acceleration: e2B
				$\frac{\text{d[TC]}}{\text{dt}}={\text{R}}_{14}-{\text{R}}_{15}-{\text{R}}_{16}-{\text{R}}_{18}+{\text{R}}_{21}$	${\text{GTP}}_{\text{T}}=[\text{GTP}]+[{\text{C}}_{1}]+[{\text{C}}_{2}]+[{\text{C}}_{\text{3p}}]$				Brake: e5
				$\frac{\text{d[kin]}}{\text{dt}}=-{\text{R}}_{5}+{\text{R}}_{6}-{\text{R}}_{18}+{\text{R}}_{19}$	$+[{\text{C}}_{\text{4p}}]+[\text{TCkin}]+[{\text{TC}}_{\text{p}}]+[\text{TCpPP}]+[\text{TC}]+[\text{e2GTP}]$				
				$\frac{\text{d[PP]}}{\text{dt}}=-{\text{R}}_{7}+{\text{R}}_{8}-{\text{R}}_{20}+{\text{R}}_{21}$	${\text{PP}}_{\text{T}}=[\text{PP}]+[\text{TCpPP}]+[\text{e2pPP}]$				PM-SC: Competition between e5 and e2B for e2
				$\frac{\text{d}[\text{e2}]}{\text{dt}}=-{\text{R}}_{1}$	${\text{e2B}}_{\text{T}}=[\text{e2B}]+[{\text{C}}_{2}]+[{\text{C}}_{\text{4p}}]$				Displacement of e2B by e5
				$\frac{\text{d}[\text{GDP}]}{\text{dt}}=-{\text{R}}_{1}+\text{R}12$	$+[{\text{Ce}}_{2}]+[{\text{Ce}}_{\text{4p}}]+[\text{e2e2B}]$				Acceleration: -
				$\frac{\text{d}[\text{e2GDP}]}{\text{dt}}={\text{R}}_{1}-{\text{R}}_{2}-{\text{R}}_{3}-{\text{R}}_{5}+{\text{R}}_{8}$	${\text{e5}}_{\text{T}}=[\text{e5}]+[{\text{C}}_{1}]+[{\text{C}}_{\text{3p}}]+[{\text{Ce}}_{1}]$				Brake: e2B, e5
				$\frac{\text{d}[{\text{Ce}}_{1}]}{\text{dt}}={\text{R}}_{2}-{\text{R}}_{4}$	$+[{\text{Ce}}_{\text{3p}}]$				
				$\frac{\text{d[e2kin]}}{\text{dt}}={\text{R}}_{5}-{\text{R}}_{6}$	${\text{GDP}}_{\text{T}}=[\text{GDP}]+[{\text{Ce}}_{1}]+[{\text{Ce}}_{2}]+[{\text{Ce}}_{\text{3p}}]$				SM-NC: Competition between e5 and e2B for e2
				$\frac{\text{d[e2pPP]}}{\text{dt}}={\text{R}}_{7}-{\text{R}}_{8}$	$+[{\text{Ce}}_{\text{4p}}]+[\text{e2GDP}]+[\text{e2kin}]+[\text{e2pGDP}]+[\text{e2pPP}]$				Displacement of e2B by e5
				$\frac{\text{d[e2pGDP]}}{\text{dt}}={\text{R}}_{6}-{\text{R}}_{7}-{\text{R}}_{9}-{\text{R}}_{10}$	${\text{e2}}_{\text{T}}=[\text{e2}]+[{\text{C}}_{1}]+[{\text{C}}_{2}]+[{\text{C}}_{\text{3p}}]$				Acceleration: e5
				$\frac{\text{d}[{\text{Ce}}_{\text{4p}}]}{\text{dt}}={\text{R}}_{10}+{\text{R}}_{11}$	$+[{\text{C}}_{\text{4p}}]+[{\text{Ce}}_{1}]+[{\text{Ce}}_{2}]+[{\text{Ce}}_{\text{3p}}]+[{\text{Ce}}_{\text{4p}}]$				Brake: e2B
				$\frac{\text{d}[{\text{Ce}}_{\text{3p}}]}{\text{dt}}={\text{R}}_{9}-{\text{R}}_{11}$	$+[\text{TCkin}]+[{\text{TC}}_{\text{p}}]+[\text{TCpPP}]+[\text{TC}]+[\text{e2}]+[\text{e2GTP}]$				
				$\frac{\text{d}[{\text{C}}_{1}]}{\text{dt}}={\text{R}}_{15}+{\text{R}}_{17}$	+[e2e2B]+[e2kin]+[e2pGDP]+[e2pPP]				SM-SC: Competition between e5 and e2B for e2
				$\frac{\text{d}[{\text{C}}_{2}]}{\text{dt}}={\text{R}}_{16}-{\text{R}}_{17}$					Displacement of e5 by e2B
				$\frac{\text{d[TCkin]}}{\text{dt}}={\text{R}}_{18}-{\text{R}}_{19}$					Acceleration: -
				$\frac{\text{d[TCpPP]}}{\text{dt}}={\text{R}}_{20}-{\text{R}}_{21}$					Brake: e2B, e5
				$\frac{\text{d}[{\text{TC}}_{\text{p}}]}{\text{dt}}={\text{R}}_{19}-{\text{R}}_{20}-{\text{R}}_{22}-{\text{R}}_{23}$					
				$\frac{\text{d}[{\text{C}}_{\text{4p}}]}{\text{dt}}={\text{R}}_{23}+{\text{R}}_{24}$					
				$\frac{\text{d}[{\text{C}}_{\text{3p}}]}{\text{dt}}=\text{R}22-{\text{R}}_{24}$					
				$\frac{\text{d[GTP]}}{\text{dt}}=-\text{R}13$					
				$\frac{\text{d[tR]}}{\text{dt}}=-{\text{R}}_{14}$					
				$\frac{\text{d[e2e2B]}}{\text{dt}}=\text{R}12-\text{R}13$					
				$\frac{\text{d[e2GTP]}}{\text{dt}}=\text{R}13-\text{R}14$					

In the PM network, CRNT reveals eight complexes and four linkage classes; the rank is three. The network’s deficiency is one. The deficiency one report of CRNToolbox confirms that the network cannot admit multiple positive steady states. Therefore, we want to know the nature of dynamics in the PM and whether the network exhibits a graded or ultrasensitive response since bistability is ruled out. For the choice of parameters, we first determine the influence of total e5 (e5_T_) on the formation of complex Ce_2_, the final output of the PM.

We perform steady state analysis using XPPAUT [10] with total e2B (e2B_T_) as the bifurcation parameter against Ce_2_ (Fig 1B). The parameters used are given in Table 2, and the parameter estimation details are provided in the Supporting information S14 Supplementary.pdf. The plots are generated using MATLAB [11].

We sweep e2B_T_ in the range of 1-1000 nM for different values of e5_T_. For e5_T_, we took the concentration in the range 0 < e5_T_ < 100, and the network exhibits both graded and ultrasensitive responses. The ultrasensitivity is present only for a narrow range of e5_T_ values (20-100 nM). To confirm both graded (RC < 1) and ultrasensitivity responses (RC > 1), we plot the response coefficient (Fig 1C). We confirm that the competition reaction, along with the displacement reaction, gives rise to ultrasensitivity. However, the range is narrow, and we see ultrasensitivity only for a certain ratio of $\frac{e5_{T}}{e2B_{T}}$. This indicates that the competition between these two species exhibits ultrasensitivity at a certain ratio, or otherwise, the model exhibits only a graded response.

**Table 2:** Kinetic parameters.

Reactions	ISR (Fitted parameters) Fig 6			Source	Reference	Tristability range				Parameters used					
						ISR				Normal Conditions Fig 2F			ISR		
						kf		kb					Primary Mechanism Fig 3B		
	kf	kb	Keq = kf/kb			lower	upper	lower	upper	kf	kb	Keq = kf/kb	kf	kb	Keq = kf/kb
**R1**	1.73 ${\text{nM}}^{-1}{\text{s}}^{-1}$	0.3156 ${\text{s}}^{-1}$	5.48 ${\text{nM}}^{-1}$	Time series	[12–14]	1E-07	10000	0	10000	0.00013	0.3156	0.0004	1.73	0.3156	5.481
**R2**	0.0006 ${\text{nM}}^{-1}{\text{s}}^{-1}$	0.7067 ${\text{s}}^{-1}$	0.0008 ${\text{nM}}^{-1}$	Time series	[12–14]	0	0.79	0.532	1.3	0.0006	0.7067	0.0008	0.0006	0.7067	0.0008
**R3**	18.06 ${\text{nM}}^{-1}{\text{s}}^{-1}$	483.3 ${\text{s}}^{-1}$	0.0374 ${\text{nM}}^{-1}$	Steady state	[2]	10.91	58.29	135	546.9	18.06	483.31	0.0373	18.06	483.31	0.0374
**R4**	1.21 ${\text{nM}}^{-1}{\text{s}}^{-1}$	727.08 ${\text{nM}}^{-1}{\text{s}}^{-1}$	0.0017	Time series	[12–14]	0.6013	1.61	623	1326	1.21	727.08	0.0017	1.21	727.08	0.0017
**R5**	2.68 ${\text{nM}}^{-1}{\text{s}}^{-1}$	5.51 ${\text{s}}^{-1}$	0.4864 ${\text{nM}}^{-1}$	Time series	[12–14]	0.83	4.387	1.064	30.73	-	-	-	2.68	5.51	0.4864
**R6**	5.82 ${\text{s}}^{-1}$	-	-	Time series	[12–14]	1.078	32.1	-	-	-	-	-	5.82	-	-
**R7**	19.05 ${\text{nM}}^{-1}{\text{s}}^{-1}$	0.0979 ${\text{s}}^{-1}$	194.59 ${\text{nM}}^{-1}$	Time series	[12–14]	14.56	10000	0	0.1325	-	-	-	19.05	0.0979	194.59
**R8**	0.025 ${\text{s}}^{-1}$	-	-	Time series	[12–14]	0.01304	0.078	-	-	-	-	-	500	-	-
**R9**	28.51 ${\text{nM}}^{-1}{\text{s}}^{-1}$	0.0233 ${\text{s}}^{-1}$	1223.6 ${\text{nM}}^{-1}$	Time series	[12–14]	0	39.65	0	10000	-	-	-	28.51	0.0233	1223.6
**R10**	20 ${\text{nM}}^{-1}{\text{s}}^{-1}$	80 ${\text{s}}^{-1}$	0.25 ${\text{nM}}^{-1}$	Steady state	[2]	0	103.5	57.82	10000	-	-	-	20	80	0.25
**R11**	297.82 ${\text{nM}}^{-1}{\text{s}}^{-1}$	440.86 ${\text{nM}}^{-1}{\text{s}}^{-1}$	0.6755	Time series	[12–14]	218.5	10000	0	610	-	-	-	297.82	80	3.72
**R12**	46.07 ${\text{nM}}^{-1}{\text{s}}^{-1}$	0.4272 ${\text{s}}^{-1}$	107.84 ${\text{nM}}^{-1}$	Time series	[12–14]	18.67	150.304	0.1316	0.696	46.07	0.4272	107.84	-	-	-
**R13**	0.433 ${\text{nM}}^{-1}{\text{s}}^{-1}$	0.0105 ${\text{nM}}^{-1}{\text{s}}^{-1}$	41.24	Time series	[12–14]	0.2653	1.41244	0.0033	0.01731	0.433	1000	0.0004	-	-	-
**R14**	1 ${\text{nM}}^{-1}{\text{s}}^{-1}$	23.7 ${\text{s}}^{-1}$	0.0422 ${\text{nM}}^{-1}$	Steady state	[15]	0.689	2.112	11.24	34.57	1	23.7	0.0422	**Secondary mechanism Fig 3E**		
**R15**	21.25 ${\text{nM}}^{-1}{\text{s}}^{-1}$	1.19 ${\text{s}}^{-1}$	17.86 ${\text{nM}}^{-1}$	Time series	[12–14]	8.112	23.89	1.081	1.689	0.06	1.19	0.0504	0.06	1.19	0.0504
**R16**	15.15 ${\text{nM}}^{-1}{\text{s}}^{-1}$	673.58 ${\text{s}}^{-1}$	0.0225 ${\text{nM}}^{-1}$	Steady state	[2]	9.656	32.41	0	1263	15.15	673.58	0.0225	15.15	673.58	0.0225
**R17**	1139.9 ${\text{nM}}^{-1}{\text{s}}^{-1}$	0.3841 ${\text{nM}}^{-1}{\text{s}}^{-1}$	2967.72	Time series	[12–14]	587	10000	0	0.6645	1139.9	0.3841	2967.72	1139.9	0.3841	2967.72
**R18**	4.34 ${\text{nM}}^{-1}{\text{s}}^{-1}$	6.52 ${\text{s}}^{-1}$	0.6656 ${\text{nM}}^{-1}$	Time series	[12–14]	4	6.77	1.748	8.143	-	-	-	4.34	6.52	0.6656
**R19**	6.78 ${\text{s}}^{-1}$	-	-	Time series	[12–14]	3.385	9.5	-	-	-	-	-	6.78	-	-
**R20**	3.23 ${\text{nM}}^{-1}{\text{s}}^{-1}$	0.0183 ${\text{s}}^{-1}$	176.5 ${\text{nM}}^{-1}$	Time series	[12–14]	0.3	4.3	0	712	-	-	-	3.23	0.0183	176.5
**R21**	71.84 ${\text{s}}^{-1}$	-	-	Time series	[12–14]	35.34	10000	-	-	-	-	-	71.84	-	-
**R22**	13.94 ${\text{nM}}^{-1}{\text{s}}^{-1}$	0.7179 ${\text{s}}^{-1}$	19.42 ${\text{nM}}^{-1}$	Time series	[12–14]	12.37	24	0	127.9	-	-	-	13.94	0.7179	19.42
**R23**	6 ${\text{nM}}^{-1}{\text{s}}^{-1}$	100 ${\text{s}}^{-1}$	0.06 ${\text{nM}}^{-1}$	Steady state	[2]	0	9.434	77.19	111	-	-	-	6	100	0.06
**R24**	6457.02 ${\text{nM}}^{-1}{\text{s}}^{-1}$	114.89 ${\text{nM}}^{-1}{\text{s}}^{-1}$	56.2	Time series	[12–14]	2246	10000	0	394.7	-	-	-	6457.02	114.89	56.2

## Normal conditions: Only graded response results in SM due to competition and displacement

Similar to the analysis done on PM under normal conditions, we perform the numerical analysis for the SM network. It can be seen from the reaction network that the reactions in both mechanisms mirror each other, though the species are different. For example, we can map e2GDP in PM to TC in SM. Similarly, we can map Ce1 and Ce2 in PM to C1 and C2 in SM. Therefore, this leads to mapping reactions *R*2, 3, 4 in PM to *R*15, 16, 17 in SM.

In SM, the three reactions in the network (Fig 1D): R15–R17, captures the competition between e5 and e2B for TC that forms complexes C_1_ and C_2_, respectively. The e5 competition delays the e2B binding to TC, and e5 displaces the e2B from the complex C_2_ to form the complex C_1_. We believe competition and displacement may bring in threshold and ultrasensitivity dynamics similar to PM. The corresponding ODEs and the mass conservation relations are provided in Table 1 (row 2). To check whether the network can exhibit bistable dynamics, we perform the CRNT analysis for the SM network.

In the SM network, CRNT reveals six complexes and three linkage classes; the rank is two. The network’s deficiency is one. The deficiency one report of CRNToolbox confirms that the network cannot admit multiple positive steady states. Since the bistability is ruled out, we want to know whether the SM network exhibits a graded or ultrasensitive response. For the choice of parameters, we determine the influence of total e5 (e5_T_) on the formation of complex C_2_.

We perform the steady state analysis using XPPAUT [10] with total e2B (e2B_T_) as the bifurcation parameter against C_2_ (Fig 1E). We sweep e2B_T_ in the range of 1–1000 nM for different values of e5_T_. For e5_T_, we took the concentration in the range 0 < e5_T_ < 100, and the network exhibits only graded responses. To confirm the graded (RC < 1) response, we plot the response coefficient (Fig 1F). We confirm that the competition reaction, along with the displacement reaction, gives rise to a graded response in the secondary mechanism.

## Occurrence of bistability due to implicit positive feedback in the model of PM (M1) and SM (M2) coupled by GTP-GDP exchange/Met-tRNA (M3) under normal conditions

Since PM and SM exhibit ultrasensitivity and graded response, respectively, we want to know whether adding GDP-GTP exchange to couple PM and SM will exhibit any new dynamics. The full circuit is shown in Fig 2A. In this mechanism, we have 10 reactions overall. The corresponding ODEs that capture PM, SM, and the GDP-GTP exchange/Met-tRNA reactions are provided in Table 1 (row 3).

We performed a structural analysis of the network using the CRNToolbox, which revealed 19 complexes and nine linkage classes, and the rank was found to be 8. The network deficiency is two and indicates the presence of bistability in this network. This indicates that adding GDP-GTP exchange with Met-tRNA addition gives rise to implicit positive feedback in the network. To generate bistability, we construct a bifurcation diagram for which we took the parameters as given in Table 2.

We construct two bifurcation diagrams: (i) to determine how the ratio of $\frac{{\text{e5}}_{\text{T}}}{{\text{e2B}}_{\text{T}}}$ affects bistability, and (ii) how coupling PM and SM by GDP-GTP and tR causes bistability. We showed earlier that PM and SM alone cannot exhibit bistability, and therefore, we want to know how GDP-GTP exchange/Met-tRNA reactions contribute to bistability.

To illustrate (i), we construct a bifurcation diagram with e2B_T_ as a bifurcation parameter against e2GTP for a fixed value of e5_T_ and GDP-GTP ratio. We track the e2GTP as the threshold indicator level for the commencement of translation initiation. In Fig 2B, the level of e2GTP is flat for a certain level of e2B_T_, after which, with its increase, the e2GTP level jumps to a new level, indicating the initiation process. Further increase of e2B_T_ leads to a gradual decrease of e2GTP, signifying the completion of initiation. Correspondingly, TC also rises and falls with a delay (Fig 2C). The model exhibits biphasic but not bistable dynamics. We reason it as follows. Initially, there is a very low e2GTP formation (Fig 2B) due to e5 that prevents the formation of PIC complex e2GTP. When a stoichiometric amount of e2B competes with e5, initiation happens with e2GTP transitioning from a lower to a higher steady state (Fig 2B). At a higher steady state of e2GTP, tR binds to form a ternary complex that results in TC increasing (Fig 2C), and the release of e2B takes place. However, e2B release results in its immediate binding to TC to form a tetramer complex C_2_, reducing the level of TC. We capture this rise and fall as a biphasic dynamics (Figs 2B and 2C). We believe it can only occur for certain tight $\frac{e5_{T}}{e2B_{T}}$ ratios to get bistable dynamics. Therefore, we took different $\frac{e5_{T}}{e2B_{T}}$ ratios to determine the occurrence of bistable dynamics (Fig 2D), which we detail in the subsequent section. We also observe an increase in threshold with an increase in e5_T_ concentration, which keeps e2GTP below the subthreshold value, thereby delaying the commencement of the initiation process. This threshold increase is because of the competition by e5 for e2GDP that disallows the binding of e2B to e2GDP for the formation of e2GTP and TC. Once e2B is in excess, it displaces e5, and the e2GTP transition from the subthreshold level occurs, leading to ultrasensitivity. In Fig 2E, we show the magnitude of ultrasensitivity by plotting the response coefficient.

We show bistability in Fig 2F with two reversible saddle-node (SN) bifurcations of e2GTP with e2B_T_ as the parameter. Unlike ultrasensitivity, translation initiation is a discontinuous transition with e2GTP switches from a subthreshold steady state to a higher steady state (SN1, Fig 2F). Once the translation initiation is complete, TC is dismantled, e2B is reduced due to binding with e2GDP, e5 competes with e2B for e2GDP, and the process gets ready for the next round of the initiation process. However, if the conditions are unfavorable, a second SN bifurcation point leads to translation termination. For example, if e2B_T_ availability is less due to mutation or other factors, then the transition from initiation to termination occurs by a second SN bifurcation immediately (SN2, Fig 2F). It is unclear which of these processes initiates or terminates the translation, and the model captures both scenarios.

To illustrate (ii), again, we believe that the coupling between the PM and SM by GDP-GTP exchange/Met-tRNA causes bistability only for a certain ratio of $\frac{e5_{T}}{e2B_{T}}$. As we showed in Fig 1A–1F, in the absence of coupling, in both PM and SM, the model exhibits only ultrasensitivity, and bistability is absent. In Fig 2G–2I, we show the bifurcation diagram with total GTP (GTP_T_) as the bifurcation parameter against TC. We took three ratios of $\frac{e5_{T}}{e2B_{T}}$, and the transition from ultrasensitivity to bistability to a graded response occurs. However, bistability happens at a small parameter range, indicating that the GTP_T_ and $\frac{e5_{T}}{e2B_{T}}$ tightly control the translation initiation process.

To summarize this section, we covered two main scenarios influencing the translation initiation process. The initiation can be ultrasensitive or a discontinuous jump, and the process depends strongly on the ratios of $\frac{e5_{T}}{e2B_{T}}$ or the $\frac{GDP}{GTP}$. In the subsequent sections, we extend our translation initiation model under normal conditions to capture the dynamics under the ISR conditions where translation attenuation occurs. PdP reactions with e2GDP and TC are vital in the translation attenuation process. Therefore, we construct the reaction steps involving PdP reactions based on the network built from the experimental data for both PM and SM. We follow it up with the numerical analysis of ODE using the bifurcation theory to show how attenuation differs in dynamics from normal conditions and interpret the results to provide a biological meaning.

## Fail-safe mechanisms: Invocation of ISR

ISR refers to initiating translation attenuating signalling pathways by the eIF2α kinases. Many kinases bring distinct ISR, including amino acid deficiency and mitochondrial stress. Translation attenuation is a fail-safe mechanism rapidly deployed to arrest the translation process through PdP reactions, keeping only the important downstream eukaryotic initiation factors functioning.

Two fail-safe mechanisms prevent the translation process under stressful conditions. In this work, we take the phosphorylated forms of eIF2 in PM and SM mechanisms, elucidated based on the experiments of Jennings et al., [2]. (i) In PM, phosphorylation of e2GDP prevents GDP-GTP exchange with e2B as a primary fail-safe mechanism or a pre-initiation fail-safe mechanism. (ii) In SM, phosphorylation of the TC allows e2B to dismantle the TC and releases the e5 factor as the secondary fail-safe mechanism. Below, we first write the chemical steps of PdP reactions of PM, the first fail-safe mechanism invoked under ISR to perform structural analysis using CRNT, and from which we try to gain some understanding of the dynamic properties of translation recovery and attenuation of the network under stressful conditions.

### PdP reactions in PM: A first fail-safe mechanism invoked under ISR

In PM, the translation process involves all the reactions before the GDP/GTP exchange and the formation of the TC under normal conditions. We take the same reactions R1 - R4 from the M1. To these reactions, we include PdP reactions to explain the fail-safe mechanism under ISR.

In PM, the PdP reactions start with kinase-phosphatase phosphorylating-dephosphorylating the dimer e2GDP. The reactions R5 and R6 below capture the reversible phosphorylation reaction, while R7 and R8 represent the dephosphorylation reactions occurring in the α subunit of e2GDP. We represent the phosphorylation reaction catalysed by the enzyme kinase (kin) [16] and the dephosphorylation reaction catalysed by phosphatase (PP). The PdP reactions are written as two-step Michaelis-Menten (MM) kinetics reactions, where the first step forms a complex, and the second step produces the phosphorylated product and releases the enzyme. The MM kinetics of PdP reactions are as follows.


$$\begin{array}{llllllllllll} & \text{R5}:\; & \text{e2GDP}+\text{kin} & \overset{{\text{kf}}_{5}}{\underset{{\text{kb}}_{5}}{⇌}}\text{e2kin}\; & {\text{R}}_{5} & ={\text{kf}}_{5}[\text{e2GDP}][\text{kin}]-{\text{kb}}_{5}[\text{e2kin}]\; & & \; & & \; & & \;\\ & \text{R6}:\; & \text{e2kin} & \overset{{\text{kf}}_{6}}{\to }\text{e2pGDP}+\text{kin}\; & {\text{R}}_{6} & ={\text{kf}}_{6}[\text{e2kin}]\; & & \; & & \; & & \;\\ & \text{R7}:\; & \text{e2pGDP}+\text{PP} & \overset{{\text{kf}}_{7}}{\underset{{\text{kb}}_{7}}{⇌}}\text{e2pPP}\; & {\text{R}}_{7} & ={\text{kf}}_{7}[\text{e2pGDP}][\text{PP}]-{\text{kb}}_{7}[\text{e2pPP}]\; & & \; & & \; & & \;\\ & \text{R8}:\; & \text{e2pPP} & \overset{{\text{kf}}_{8}}{\to }\text{e2GDP}+\text{PP}\; & {\text{R}}_{8} & ={\text{kf}}_{8}[\text{e2pPP}]\; & & \; & & \; & & \; \end{array}$$

The next couple of reactions, R9-R10, capture the competitive reactions of e5 and e2B. They compete for the phosphorylated e2GDP (e2pGDP) obtained from the earlier reactions. The complex e2pGDP has multiple subunits, and e5 and e2B bind to different subunits, affecting the competitive process. For example, e2pGDP has four subunits; αβγδ. In the reaction R9 below, e5 binds with the β and γ subunits of e2pGDP to form a complex Ce_3p_ [2], while in the reaction R10, e2B binds to the α subunit of e2pGDP regulatory site [6,8] to form the stable complex Ce_4p_ [4]. The binding of the phosphorylated site by e5 restricts the GEF activity of e2B, disallowing translation initiation.


$$\begin{array}{llllllllllll} & \text{R9}:\; & \text{e2pGDP}+\text{e5} & \overset{{\text{kf}}_{9}}{\underset{{\text{kb}}_{9}}{⇌}}{\text{Ce}}_{\text{3p}}\; & {\text{R}}_{9} & ={\text{kf}}_{9}[\text{e2pGDP}][\text{e2B}]-{\text{kb}}_{9}[{\text{Ce}}_{\text{3p}}]\; & & \; & & \; & & \;\\ & \text{R10}:\; & \text{e2pGDP}+\text{e2B} & \overset{{\text{kf}}_{10}}{\underset{{\text{kb}}_{10}}{⇌}}{\text{Ce}}_{\text{4p}}\; & {\text{R}}_{10} & ={\text{kf}}_{10}[\text{e2pGDP}][\text{e2B}]-{\text{kb}}_{10}[{\text{Ce}}_{\text{4p}}]\; & & \; & & \; & & \; \end{array}$$

From the experiments of Jennings et al., [2], we also know that the e2B antagonises the Ce_3p_ and displaces e5 to form the complex Ce_4p_. Below, R11 captures this reaction.


$$\begin{array}{llllllllllll} & \text{R11}:\; & {\text{Ce}}_{\text{3p}}+\text{e2B} & \overset{{\text{kf}}_{11}}{\underset{{\text{kb}}_{11}}{⇌}}{\text{Ce}}_{\text{4p}}+\text{e5}\; & {\text{R}}_{11} & ={\text{kf}}_{11}[{\text{Ce}}_{\text{3p}}][\text{e2B}]-{\text{kb}}_{11}[{\text{Ce}}_{\text{4p}}][\text{e5}]\; & & \; & & \; & & \; \end{array}$$

To summarise, the reactions R5 - R11 capture the complete set of PdP reactions and the competitive reactions of e5 and e2B with e2pGDP. To these reactions, we include all the reactions we built in M1. This comprises the PM of the ISR network. We provide the corresponding differential equations in Table 1 (row 4).

Structural analysis by CRNT reveals twenty complexes and nine linkage classes, and the rank is eight. The deficiency of the network is 3. CRNT shows the presence of bistability in this network. We also note that the PM, in the absence of PdP reaction, exhibited either graded or ultrasensitivity response (Fig 1A–1C). However, CRNT analysis indicates that adding PdP reactions in ISR generates bistability.

### Interpretation of bistability in ISR’s PM: Attenuation and recovery of translation is a memory process

**Fig 3:**
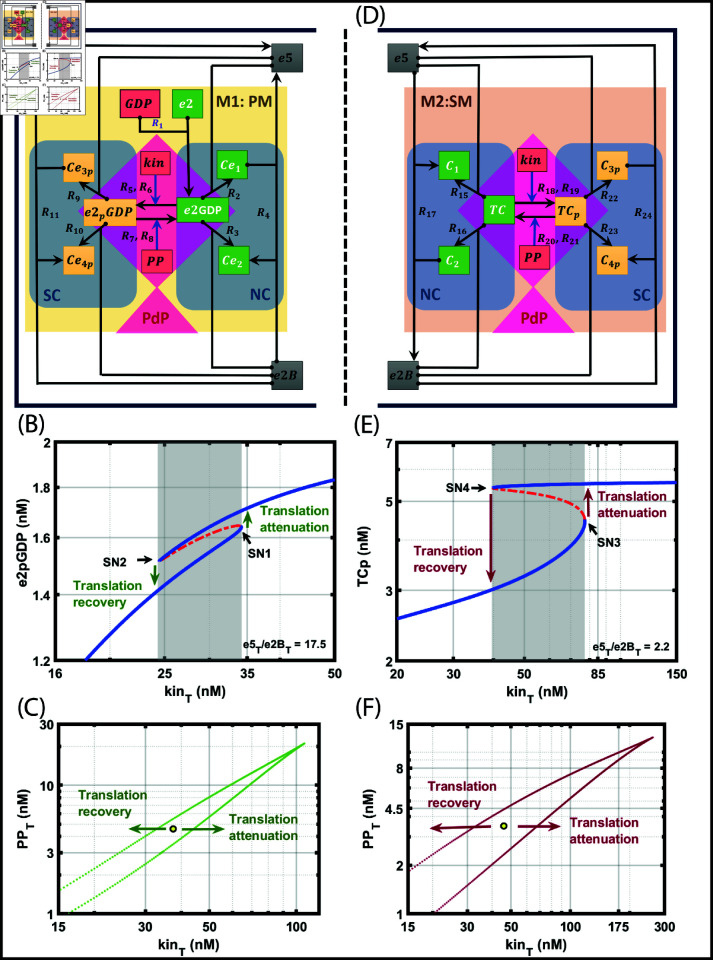
Module 1 (M1), the primary mechanism (PM), and Module 2 (M2), the secondary mechanism (SM) under normal (NC) and stressful (SC) conditions along with the phosphorylation and dephosphorylation reactions (PdP). A: Primary mechanism of translation initiation under normal and stressful conditions. This network captures all the interactions involving e5 and e2B with e2 and the phosphorylation and dephosphorylation reactions. B: Bifurcation diagram of e2pGDP with total kinase (kin_T_) as the parameter. The level of kin_T_ represents the amount of stress. With the increase in kin_T_ concentration, the concentration of e2pGDP switches from lower to higher, which indicates translation attenuation. In this context, the two bistable states correspond to translation attenuation and recovery. The total concentrations of free e2, e2B, e5, PP, and GDP were kept constant at 55, 2, 35, 3, and 1260 nM, respectively. C: Stress-destress plane, kin_T_ and PP_T_, are the two parameters used to show the attenuation and recovery of translation. The region within the green line represents the bistable region. The yellow dot represents the neutral state of the checkpoint. As the arrows point in the diagram, the checkpoint is engaged towards the right of the neutral checkpoint, and on the left, the checkpoint is disengaged. The activation and deactivation correspond to translation attenuation and recovery, respectively. D: Secondary mechanism of translation initiation under normal and stressful conditions. This network captures all the interactions involving e5 and e2B with TC and the phosphorylation and dephosphorylation reactions. E: Bifurcation diagram of TCp with total kinase (kin_T_) as the parameter. The two bistable states correspond to translation attenuation and recovery. The total concentrations of free TC, e2B, e5, and PP were kept constant at 50, 18, 40, and 4 nM, respectively. F: Stress-destress plane, kin_T_ and PP_T_, are the two parameters used to show the attenuation and recovery of translation. The XPPAUT files used for simulations is in the Supporting information S10 Fig3A.ode, and S11 Fig3D.ode.

To show the presence of bistability, we construct a bifurcation diagram with total kinase (kin_T_) as the bifurcation parameter against e2pGDP (Fig 3B) as the output response. We maintain the ratio $\frac{{\text{e5}}_{\text{T}}}{{\text{e2B}}_{\text{T}}}=17.5$. The kin_T_ is proportional to the stress present in the network. With the increase in kin_T_ concentration, the concentration of e2pGDP exhibits ultrasensitivity (Fig 3B). At $kin_{T}\approx 34.19$ nM, the system crosses the first SN bifurcation (SN1), resulting in a switch from a lower to a higher steady state. Similarly, when kin_T_ level decreases due to destress, at $kin_{T}\approx 24.46$ nM, the system crosses a second SN bifurcation point (SN2), where there is a jump from a higher to a lower steady state. We attribute the lower levels of kin_T_-e2pGDP in the bifurcation diagram to translation recovery since the stress is low or insignificant, and most of the e2GDP is freely available for further reactions other than the phosphorylation reactions. When stress increases, kin_T_ proportionately increases and exceeds the threshold, an SN bifurcation point, where all the e2GDPs get phosphorylated, and translation attenuation happens. Our model’s two dynamical steady states correspond to a translation attenuation (upper steady state) and a recovery (lower steady state). The crossing of SN bifurcations on either side indicates a rapid switch of recovery or attenuation, but it’s also history-dependent. Importantly, our PM model captures the **reversible** transition between the two steady states. This is because the translation does not completely stop all the processes under stress but only delays the recovery. However, translation recovery should happen instantaneously once it retains normalcy and is destressed, and the second SN in the model captures the rapid switch from attenuation to recovery.

We also construct a two-parameter bifurcation diagram in the kin_T_ vs. PP_T_ plane, which can be thought of as a stress-destress or attenuation-recovery plane (Fig 3C). The model of phosphorylated-PM exhibits cusp-bifurcation in a narrow region. We marked the region with a circle inside the cusp where, under stress, three steady states, namely low, intermediate, and high e2pGDP, can coexist, indicating that it is history-dependent. For example, when $\frac{kin_{T}}{PP_{T}}$ is high, attenuation occurs as the system moves to the right from the circle, crosses the boundary, and becomes monostable. If the ratio is low, recovery occurs, the system moves to the left of the circle, crosses the boundary, and becomes monostable.

This raises the question of the role of the phosphorylated-SM, which operates as a second fail-safe mechanism. To infer SM’s role, in the subsequent section, we go through the same process as in phosphorylated-PM: writing all the PdP reactions for TC, then coupling with PM, writing ODEs, and performing structural and dynamical analysis. We find that the reactions of phosphorylated-SM mirror the reactions of phosphorylated-PM.

### PdP reactions of SM: The second fail-safe mechanism

The second fail-safe mechanism represents a route for e2B to capture additional forms of e2. For example, in SM, the phosphorylated e2 in the form of TC or TC-containing complexes will be the substrate for e2B for fail-safe control. This is the secondary fail-safe mechanism for ISR. The PdP reactions of SM involve TC, and the reactions R18 - R21 capture it below. Again, we write the PdP reactions as a two-step, slow-fast MM kinetics. We retain all the notations for the enzymes kinase and phosphatase; TC is the substrate.


$$\begin{array}{llllllllllll} & \text{R18}:\; & \text{TC}+\text{kin} & \overset{{\text{kf}}_{18}}{\underset{{\text{kb}}_{18}}{⇌}}\text{TCkin}\; & {\text{R}}_{18} & ={\text{kf}}_{18}[\text{TC}][\text{kin}]-{\text{kb}}_{18}[\text{TCkin}]\; & & \; & & \; & & \;\\ & \text{R19}:\; & \text{TCkin} & \overset{{\text{kf}}_{19}}{\to }{\text{TC}}_{\text{p}}+\text{kin}\; & {\text{R}}_{19} & ={\text{kf}}_{19}[\text{TCkin}]\; & & \; & & \; & & \;\\ & \text{R20}:\; & {\text{TC}}_{\text{p}}+\text{PP} & \overset{{\text{kf}}_{20}}{\underset{{\text{kb}}_{20}}{⇌}}\text{TCpPP}\; & {\text{R}}_{20} & ={\text{kf}}_{20}[{\text{TC}}_{\text{p}}][\text{PP}]-{\text{kb}}_{20}[\text{TCpPP}]\; & & \; & & \; & & \;\\ & \text{R21}:\; & \text{TCpPP} & \overset{{\text{kf}}_{21}}{\to }\text{TC}+\text{PP}\; & {\text{R}}_{21} & ={\text{kf}}_{21}[\text{TCpPP}]\; & & \; & & \; & & \; \end{array}$$

Again, as in PM, e5 and e2B compete for the phosphorylated TC (TC_p_). TC_p_ binds with e5 to form a complex C_3p_, but experimentally, it was shown that the complex C_3p_ is very unstable, and e2B can easily disrupt and release e5 immediately [2]. This process prevents the initiation of translation. Similarly, TC_p_ binds with e2B to form a stable complex C_4p_, since TC_p_ inhibits e2B’s GEF activity [3,8] by tightly binding to it [6,8,17]. The reactions R22 - R23 given below capture the competitive reactions.


$$\begin{array}{llllllllllll} & \text{R22}:\; & {\text{TC}}_{\text{p}}+\text{e5} & \overset{{\text{kf}}_{22}}{\underset{{\text{kb}}_{22}}{⇌}}{\text{C}}_{\text{3p}}\; & {\text{R}}_{22} & ={\text{kf}}_{22}[{\text{TC}}_{\text{p}}][\text{e5}]-{\text{kb}}_{22}[{\text{C}}_{\text{3p}}]\; & & \; & & \; & & \;\\ & \text{R23}:\; & {\text{TC}}_{\text{p}}+\text{e2B} & \overset{{\text{kf}}_{23}}{\underset{{\text{kb}}_{23}}{⇌}}{\text{C}}_{\text{4p}}\; & {\text{R}}_{23} & ={\text{kf}}_{23}[{\text{TC}}_{\text{p}}][\text{e2B}]-{\text{kb}}_{23}[{\text{C}}_{\text{4p}}]\; & & \; & & \; & & \; \end{array}$$

Finally, since the ternary complex C_3p_ is unstable, e2B can bind and disrupt the complex to release e5 [2]. We write it in the reaction R24 below, where e2B binds complex C_3p_ to release e5 to produce more C_4p_.


$$\begin{array}{llllllllllll} & \text{R24}:\; & {\text{C}}_{\text{3p}}+\text{e2B} & \overset{{\text{kf}}_{24}}{\underset{{\text{kb}}_{24}}{⇌}}{\text{C}}_{\text{4p}}+\text{e5}\; & {\text{R}}_{24} & ={\text{kf}}_{24}[{\text{C}}_{\text{3p}}][\text{e2B}]-{\text{kb}}_{24}[{\text{C}}_{\text{4p}}][\text{e5}]\; & & \; & & \; & & \; \end{array}$$

Structural analysis by CRNT reveals eighteen complexes and eight linkage classes, and the rank is seven. The deficiency of the network is 3. CRNT shows the presence of bistability in this network. Similar to that of PM, the SM, in the absence of PdP reaction, exhibited either graded or ultrasensitivity response (Fig 1D–1F). However, CRNT analysis indicates that adding PdP reactions in ISR generates bistability.

Similar to PM and SM under normal conditions, which mirror each other, the PM and SM in ISR also mirror each other. We can map the species of PM and SM as follows. The species e2GDP, $Ce_{1}$, and $Ce_{2}$ in PM can be mapped to TC, $C_{1}$, and $C_{2}$ in SM. These are the same mapping we did earlier in the normal mechanism. The others are the phosphorylated species which are $e2_{p}GDP$, $Ce_{3p}$, and $Ce_{4p}$ in PM can be mapped to $TC_{p}$, $C_{3p}$, and $C_{4p}$ in SM. The kinetic parameters were taken as given in Table 2.

### Interpretation of bistability in ISR’s SM: Attenuation and recovery of translation is a memory process

To show the presence of bistability, we construct a bifurcation diagram with total kinase (kin_T_) as the bifurcation parameter against e2pGDP (Fig 3E) as the output response. We maintain the ratio $\frac{{\text{e5}}_{\text{T}}}{{\text{e2B}}_{\text{T}}}=2.2$. The kin_T_ is proportional to the stress present in the network. With the increase in kin_T_ concentration, the concentration of e2pGDP exhibits ultrasensitivity (Fig 3B). At ${\text{kin}}_{\text{T}}\approx 77.34$ nM, the system crosses the first SN bifurcation (SN3), resulting in a switch from a lower to a higher steady state. Similarly, when kin_T_ level decreases due to destress, at ${\text{kin}}_{\text{T}}\approx 39.76$ nM, the system crosses a second SN bifurcation point (SN4), where there is a jump from a higher to a lower steady state. We attribute the lower levels of kin_T_-e2pGDP in the bifurcation diagram to translation recovery since the stress is low or insignificant, and most of the e2GDP is freely available for further reactions other than the phosphorylation reactions. When stress increases, kin_T_ proportionately increases and exceeds the threshold, an SN bifurcation point, where all the e2GDPs get phosphorylated, and translation attenuation happens. Our model’s two dynamical steady states correspond to a translation attenuation (upper steady state) and a recovery (lower steady state). The crossing of SN bifurcations on either side indicates a rapid switch of recovery or attenuation, but it’s also history-dependent. Importantly, our SM model captures the **reversible** transition between the two steady states. This is because the translation does not completely stop all the processes under stress but only delays the recovery. However, translation recovery should happen instantaneously once it retains normalcy and is destressed, and the second SN in the model captures the rapid switch from attenuation to recovery.

We also construct a two-parameter bifurcation diagram in the kin_T_ vs. PP_T_ plane, which can be thought of as a stress-destress or attenuation-recovery plane (Fig 3F), similar to that of the stress-destress plane in PM. The model of phosphorylated-SM also exhibits cusp-bifurcation, and hence, it is interpreted in the same way as PM.

**Fig 4:**
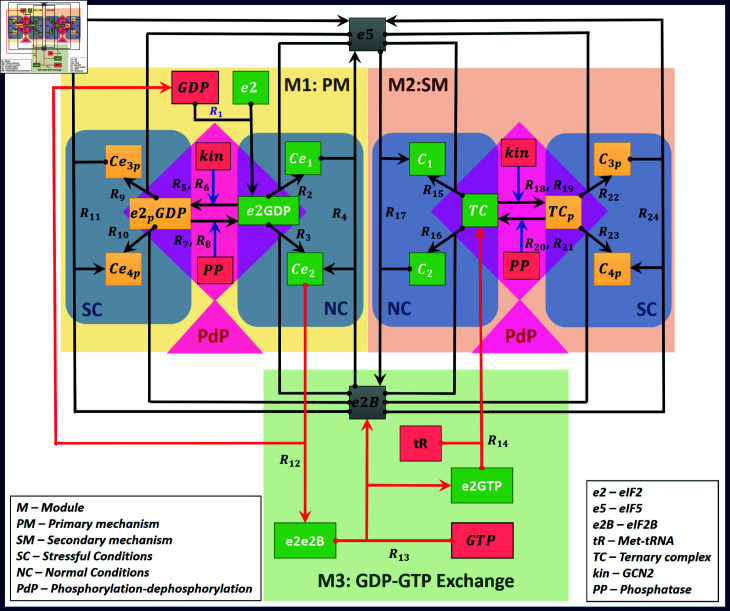
Network of integrated stress response. This circuit gives an overview of integrated stress response in the translation process. The entire network is divided into three modules: module 1 (R1-R11): primary mechanism (M1: PM), module 2 (R15-R24): secondary mechanism (M2: SM), and module 3 (R12-R14): coupling of primary and secondary mechanism by GDP-GTP exchange (M3: GDP-GTP Exchange). The reactions in M1 and M2 are further grouped into normal conditions (NC), stressful conditions (SC) and phosphorylation-dephosphorylation reactions (PdP). The phosphorylated species are shown in yellow boxes, and the unphosphorylated species in green boxes where e2 is the short form of free eIF2, and TC is the short form of the ternary complex, which is a complex of e2GTP and Met-tRNA represented by tR shown in red box. Other species, kinase (kin), phosphatase (PP), GDP, and GTP are in red boxes. The eukaryotic initiation factors eIF5 (e5) and eIF2B (e2B) are given in grey boxes. All the reactions are based on mass action kinetic laws. The blue lines represent the phosphorylation and dephosphorylation reactions. Also, the red lines are for GDP-GTP exchange and ternary complex formation. The NET file for the circuit is given in the Supporting information S6 Fig4.NET.

In summary, we provide all the steps, ODEs, and conservation relationship of three modules M1 (PM), M2 (SM), and M3 (GDP-GTP exchange that couples M1 and M2) along with PdP reactions in Fig 4 and in Table 1 (row 6). There are overall twenty-four reactions, twenty-six ODEs, and eight conservation relationships. In the subsequent section, we perform a structural analysis to determine the presence of bistability, including together all the three modules and the PdP reactions, and then we follow it up with a dynamic analysis.

Structural analysis by CRNT reveals that the network has forty-three complexes and nineteen linkage classes, and the rank (r) of the network is eighteen. The network’s deficiency is 6. The higher deficiency report of the CRNToolbox confirms that the network can admit multiple positive steady states. Therefore, we perform a bifurcation analysis with kin_T_ as the bifurcation parameter, which is proportional to the amount of stress exerted on the system.

## Effect of GTP on bistable dynamics for a constant $\frac{e5_{T}}{e2B_{T}}$ under ISR

**Fig 5:**
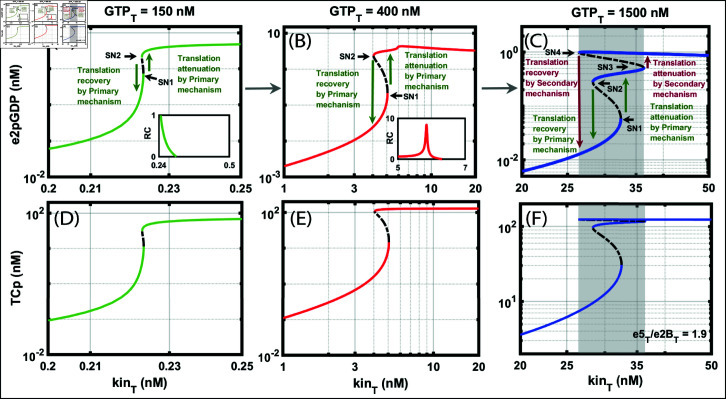
Effect of GTP on bistable dynamics for a constant $\frac{e5_{T}}{e2B_{T}}$ under ISR. A: Bifurcation diagram of e2pGDP with total kinase (kin_T_) as the parameter. We keep the total GTP (GTP_T_) at 150 nM. The inset contains the response coefficient (RC) graph. The RC graph shows a graded response. The bifurcation shows bistability. B: Bifurcation diagram of e2pGDP with kin_T_ as the parameter. We keep GTP_T_ at 400 nM. The inset contains the RC graph to show the presence of ultrasensitivity. The bifurcation shows bistability is followed by ultrasensitivity. C: Bifurcation diagram of e2pGDP with kin_T_ as the parameter. We keep GTP_T_ at 1500 nM. The bifurcation shows tristability. The first jump shown in green arrows is attributed to the translation attenuation and recovery by the primary mechanism (PM). The second jump shown in red arrows is attributed to the translation attenuation and recovery by the secondary mechanism (SM). D: Bifurcation diagram of TC_p_ with kin_T_ as the parameter. We keep GTP_T_ at 150 nM. E: Bifurcation diagram of TC_p_ with kin_T_ as the parameter. We keep GTP_T_ at 400 nM. F: Bifurcation diagram of TC_p_ with kin_T_ as the parameter. We keep GTP_T_ at 1500 nM. The bifurcation shows tristability. We keep $\frac{e5_{T}}{e2B_{T}}=1.9$. The XPPAUT file used for simulations is in the Supporting information S12 Fig4.ode.

We want to study how the GDP-GTP exchange plays a role in the translation recovery and attenuation process for which we fixed the $\frac{{\text{e5}}_{\text{T}}}{{\text{e2B}}_{\text{T}}}=1.9$. We construct the bifurcation diagram (Fig 5) with kin_T_ as the bifurcation parameter against e2pGDP and TC_p_ for three different GTP_T_. For a low value of GTP_T_ = 150 nM, the model exhibits only bistability. At an intermediate value of GTP_T_ = 400 nM, the model exhibits bistability followed by ultrasensitivity. Fig 5A, and Fig 5D show only bistability. In Fig 5B and 5E, where with an increase of kin_T_, the model exhibits bistability followed by ultrasensitivity, and we plot the response coefficient (Inset Fig 5A and 5B). Then, with a further increase of kin_T_, the model exhibits saddle-node bifurcation, SN1, approximately at $kin_{T}\approx 5$ nM. Then, it jumps to the other steady state. Similarly, when kin_T_ reduces, there is a second saddle-node bifurcation SN2, $kin_{T}\approx 4$ nM. However, when GTP_T_ is high, the coupling is stronger, and the model exhibits tristability Fig 5C and 5F, shaded region. Now, there are two jumps, one each for attenuation and recovery. We attribute the jumps from a lower to a higher steady state to translation attenuation, whereas a jump from a higher to a lower steady state to translation recovery. In the subsequent sections, we will deal in detail with the role of molecular controls responsible for this jump. We conclude this section that GTP acts as a coupler between PM and SM and that there is a threshold concentration below which the model exhibits ultrasensitivity and above which the model exhibits tristability. We will attribute tristability to PM and SM appropriately, which we will map in the subsequent sections.

## Tristable dynamics in ISR

**Fig 6:**
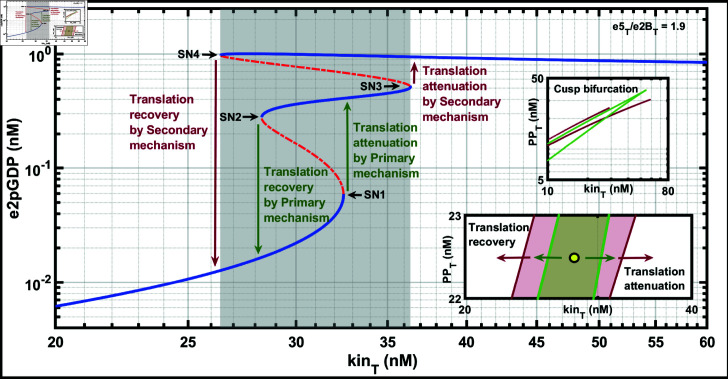
Bifurcation diagram and stress-destress plane using ISR network. Bifurcation diagram of e2pGDP with total kinase (kin_T_) as the parameter. The first jump, shown by the green arrows, represents translation attenuation and recovery by the PM. The second jump, shown by the red arrows, represents the translation attenuation and recovery by the SM. With the increase in kin_T_ concentration, the concentration of e2pGDP switches from a lower to a higher state in two jumps. However, when kin_T_ decreases with the decrease in stress, the system switches to the lowest state in one jump to start the translation process immediately. The insets show the stress-destress plane, kin_T_ and PP_T_ are the two parameters used to show the translation attenuation and recovery by PM and SM. The region within the green line represents the first jump corresponding to the PM. The region within the red line represents the second jump corresponding to the SM. The yellow dot represents the neutral state of the checkpoint. As the arrows point in the diagram, the checkpoint is engaged towards the right of the neutral checkpoint, and on the left, the checkpoint is disengaged. The translation attenuation and recovery happen in two stages: PM (green arrows) and SM (red arrows). The total concentrations of free e2, e2B, e5, PP, GTP, GDP, and tR were kept constant at 310, 53, 100, 24, 1500, 1510, and 310 nM, respectively. The XPPAUT file used for simulations is in the Supporting information S12 Fig4.ode.

To construct the bifurcation diagram, we first fixed the ratio of $\frac{{\text{e5}}_{\text{T}}}{{\text{e2B}}_{\text{T}}}=1.9$. Then, by using the parameters obtained from the parameter estimation, as given in Table 2, we construct the bifurcation diagram with kin_T_ as the bifurcation parameter against e2pGDP (Fig 6), and the model exhibits tristability. CRNT is useful in predicting the presence of bistability, but it cannot predict tristability as in the present case. The presence of tristability indicates that the translation attenuation is a multistep hysteretic process, where the attenuation takes place by a two-step bistable process when kin_T_ increases. When kin_T_ decreases, the switch can be a single-step process when there is a rapid commencement of translation recovery.

In Fig 6 insets, we show the two-parameter bifurcation of kin_T_ and PP_T_ plane to illustrate the attenuation and recovery of PM and SM under ISR. The region within the green line represents the first jump corresponding to the PM. The region within the red line represents the second jump corresponding to the SM. The yellow dot represents the kin_T_ where the stress may be optimal to hold the system neither to undergo recovery nor attenuation. Depending on the history and the amount of stress, the system may move out to either attenuation or recovery regimes in a one or two-step process. As the arrows point in the diagram, as the stress increases, for attenuation, the system moves towards the right, or otherwise, the system moves towards the left from the dot. The attenuation and recovery happen in two stages in this regime: primary (green arrows) and secondary (red arrows).

### Interpretation of tristability as a two-step translational attenuation and recovery process in ISR

Tristability is a two-step process where increased stress and kin_T_ concentration result in a gradual increase of e2pGDP. The attenuation starts after it crosses the first SN1 bifurcation point at $kin_{T}\approx 32.5$ nM. However, if the stress is transient and kin_T_ decreases, it jumps again to a low e2pGDP through the second bifurcation point SN2 at $kin_{T}\approx 28.31$ nM, and recovery continues. However, further kin_T_ increase leads to crossing the third bifurcation point SN3, $kin_{T}\approx 36.38$ nM, leading to a second, complete attenuation. Again, the system reaches the first or second attenuated steady states depending on the history of the system. According to our model, translation attenuation is a two-step process when the stress is immediate. Finally, when the stress decreases, the system returns to normalcy after it crosses the fourth bifurcation point SN4 at $kin_{T}\approx 26.41$ nM, which we attribute to translation recovery. Translation recovery from the attenuated second steady state is a one-step process; i.e., it bypasses the first bistability of PM since the recovery commences immediately.

### Mapping the tristability to phosphorylated PM and SM: The role of e5 and e2B in tristability

**Fig 7:**
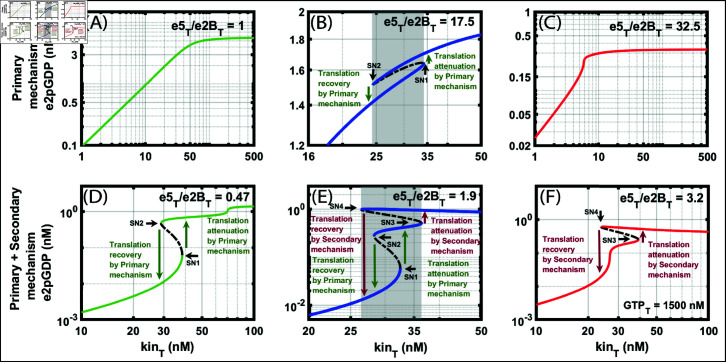
Bifurcation of e2pGDP at different ratios of $\frac{{\text{e5}}_{\text{T}}}{{\text{e2B}}_{\text{T}}}$. A: Bifurcation diagram of e2pGDP using the primary mechanism (PM) alone, with total kinase (kin_T_). We keep $\frac{{\text{e5}}_{\text{T}}}{{\text{e2B}}_{\text{T}}}=1$. B: Bifurcation diagram of e2pGDP using PM alone with kin_T_ as the parameter showing bistability. We keep $\frac{{\text{e5}}_{\text{T}}}{{\text{e2B}}_{\text{T}}}=17.5$. The jump is attributed to the translation attenuation and recovery C: Bifurcation diagram of e2pGDP using PM alone with kin_T_. We keep $\frac{{\text{e5}}_{\text{T}}}{{\text{e2B}}_{\text{T}}}=32.5$. D: Bifurcation diagram of e2pGDP using both PM and secondary mechanism (SM) with kin_T_ as the parameter showing bistability. We keep $\frac{{\text{e5}}_{\text{T}}}{{\text{e2B}}_{\text{T}}}=0.47$. E: Bifurcation diagram of e2pGDP with kin_T_ as the parameter showing tristability. We keep $\frac{{\text{e5}}_{\text{T}}}{{\text{e2B}}_{\text{T}}}=1.9$. The first jump shown in green arrows is attributed to the translation attenuation and recovery by the PM. The second jump shown in red arrows is attributed to the translation attenuation and recovery by the SM. F: Bifurcation diagram of e2pGDP using both PM and SM with kin_T_ as the parameter showing bistability. We keep $\frac{{\text{e5}}_{\text{T}}}{{\text{e2B}}_{\text{T}}}=3.2$. In all the figures, we keep the total GTP (GTP_T_) at 1500 nM. The XPPAUT files used for simulations are in the Supporting information S10 Fig3A.ode and S12 Fig4.ode.

We now map the occurrence of tristable dynamics to the molecular controls, which are the basis of phosphorylated PM and SM’s. For that, we first fix GTP_T_ = 1500 nM and vary the $\frac{e5_{T}}{e2B_{T}}$ ratio. We construct a one-parameter bifurcation diagram with kin_T_ as the bifurcation parameter against e2pGDP for PM Fig 7A–7C. We start with a ratio $\frac{e5_{T}}{e2B_{T}}=1$, and the model exhibits a graded response. When the ratio exceeds one, $\frac{e5_{T}}{e2B_{T}}=17.5$, the model exhibits bistability. The SN bifurcation occurs for kin_T_ at 24.46 to 34.19 nM. We take this as an indicator of the PM bistable range during stress. When e5 is extremely high, $\frac{e5_{T}}{e2B_{T}}=32.5$, the model exhibits only ultrasensitvity. Therefore, for the choice of parameters, the PM exhibits only ultrasensitivity or bistability or both for different ratios of $\frac{e5_{T}}{e2B_{T}}$.

Now, we construct the bifurcation diagram of the full phosphorylated ISR model (Fig 7D–7F) and compare only with the phosphorylated form of PM (M1) (Fig 7A–7C). The model exhibits bistability and ultrasensitivity for $\frac{e5_{T}}{e2B_{T}}=0.47$. For $\frac{e5_{T}}{e2B_{T}}=1.9$, the model exhibits tristability. There are now four SN bifurcations, and they are in the kin_T_ range approximately from 26.41 to 36.38 nM, and correspondingly, the e2pGDP falls approximately in the range from 0.06 to 1 nM. We break the e2pGDP SN bifurcation points into two regions. In the first region, the SN1 and SN2 for e2pGDP are in the range of 0.06 to 0.3 nM, and in the second region, the SN3 and SN4 are in the range of 0.5 to 1 nM.

## Kinetic parameters for the models

### Parameter estimation from the steady state and time series data

We estimated the parameters using the experimental steady state and time series data. We used the open software Copasi to estimate the kinetic constants from the data [18]. We fitted the steady state data using Copasi. To fit the other parameters using the time series data, we fixed the parameters obtained from the steady state before performing the fit and the rest of the parameters were fitted by fixing the upper and lower bounds. The supporting Copasi file is provided in the Supporting information S13 ISR.cps. We also provide a Supporting information S14 Supplementary.pdf where the details of parameter fittings using steady state and time series are given. We provided in Table 2 the final estimated rate constants. Also, the rate constants and the corresponding $K_{eq}$ values of all the reactions are given in Table 2.

### Range of tristability

In the tristability, as shown in Fig 6, with the increase in total kinase, the translation attenuation takes place in two jumps, and with the decrease in the total kinase, translation recovery takes place in one jump. This is possible when the kinase concentration at SN1 is less than SN3, and SN2 is greater than SN4. We were interested in looking at the range of the individual kinetic parameters that would give rise to tristability. We got the range for each parameter by plotting the two-parameter bifurcation between every kinetic parameter and the total kinase. The details of the method are given in the Supporting information S14 Supplementary.pdf, and the final range of parameters are given in Table 2.

## Other modelling studies and comparison with our model

In this section, we compare and discuss our models of normal and ISR with other published models, some of which captured not only the dynamics of translation initiation but also elongation and termination processes. Except for Strube’s [19] model, none of the models we discuss below captured the fail-safe mechanisms and ISR.

Dimelow et al.[20] have modelled the formation of multifactor complex (MFC) formation to its binding to the mRNA. They built a kinetic model for translation initiation and attempted to quantitatively determine the rate of control of the pathway based on the limited experimental data. Unlike our model, they showed the involvement of all the factors, but the interplay between eIF5 and eIF2B was not captured in detail. Importantly, their model was built to highlight the challenges they faced when estimating the parameters and provided parameter estimation methods when there is only sparse availability of experimental data. In our case, we took whatever the available kinetic parameters were, and we fixed the rest to generate bistability. We will take up in future the estimation of kinetic parameters for our model once sufficient experimental data are available.

A similar model by Tao et al. [21] captures the MFC formation and ribosome interaction. Their objective was to model the role of phosphorylation by the kinase GCN under the amino acid histidine starvation. They analysed their GCN signalling translational pathway’s robustness to internal and external perturbations. While the individual cells in the population are sensitive to external perturbations, they are robust to internal perturbations due to GCN signalling. Their steady-state analysis of the model concluded that the dynamical behaviour of the population of cells differed from the individual cells and suggested that single-cell measurements may provide better insight into understanding the translation process. Further, they showed that the dynamics were robust even when there were considerable fluctuations in the initiation factors eIF5 and eIF1. Though their network and steady-state analysis are similar to our work, they did not study the fail-safe mechanism under ISR conditions, and their objectives differ from those of our present work.

Skjondal-Bara et al. [22] modelled the translation initiation and control by phosphorylation along with the elongation process. Their delay model predicts protein production under optimal and non-optimal conditions, which include amino acid starvation, viral attacks, and stress. They also aim to predict the consumption of energy and amino acids, ribosome loading rate, and ribosome spacing. They performed stability analysis and carried out time-series simulations to understand the nature of dynamics, but they did not extend their model to study ISR.

The role of feedback in translational control, particularly at the termination process due to a premature stop codon, was captured by de-Silva et al. [23]. They have used analytical and numerical methods to understand the dynamics of their model. Their minimal model consists of translation initiation comprising the ribosome, polysomes, start codon, and protein synthesis, but it differs from the ISR regulation of translation initiation.

The model by Strube [19] captures the translation initiation process and ISR’s effect on the initiation process. Their model comprises canonical, and ATF translation processes that capture the dynamics under ISR and analyse the role of the eIF2 phosphate pathway. Their model exhibits a bistable switch. It relates the toggling between the steady states in the switch to translation recovery and translation attenuation due to an explicit positive feedback loop in their network. They took eIF2 kinase as a bifurcation parameter that increases from a very low to a high value. Our model also behaves similarly, but the feedback mechanism in our model is not explicit. It is a non-canonical positive feedback that operates in our network. Recent studies on mRNA-miRNA interaction ([24]) and the Ras signalling pathway of the fungal species *C. albicans* [25] have non-canonical feedback that shows bistability. Strube’s [19] model has different activation mechanisms of ISR by different kinases under different stress conditions. The role of eIF2B in ISR was shown by one parameter bifurcation with total eIF2 kinase (e2kin) concentration as a parameter with a range of total eIF2B (e2B_T_) concentrations. Two parameter bifurcation analyses were also shown between total eIF2 kinase and total eIF2B. Biphasic dynamics are also seen in their analysis. Though the parameters we chose are total kinase and total phosphatase, the results obtained by our model are similar to their model. However, our more detailed model explains two different fail-safe mechanisms through the PdP of PM and SM.

## Discussion and conclusion

Understanding the protein synthesis regulation at a translational level is important during normal and ISR conditions. Mutations in the initiation factors eIF2 and eIF2B or excess of ISR results in mental retardation, epileptic seizures, schizophrenia, and obesity [26]. With new biological techniques, information regarding how signalling networks in protein translation mechanisms operate has been uncovered under normal and stressful conditions. However, the network is extremely complex, and it isn’t easy to understand the role of important initiation factors that affect the translation process. Therefore, we undertook the task of modelling the dynamics of the signalling network by converting it to a set of nonlinear differential equations and analysing them using the tools of bifurcation theory. All the reactions in the system were taken based on the experimental results given in [2]. However, we do not know whether other complexes like Ce1, Ce2, Ce3p, and Ce4p in the mechanism are mono or multiple-phosphorylated.

We modelled only the translation initiation process, and for ease, we modularised the network into the PM (M1), SM (M2), and GDP-GTP/Met-tRNA (M3) and the PdP reactions. The modules and their corresponding reactions are summarised in Table 3. Under normal conditions, the structural analysis by CRNT uncovered the presence of implicit positive feedback. This generated bistable dynamics, for which we attributed the two stable steady states to translational initiation and termination. The model also captured biphasic dynamics, which we attributed to the translational initiation and completion.

**Table 3:** ISR network: modules and their corresponding reactions.

Modules		Reactions
Module 1: Primary Mechanism (M1: PM)	Normal Conditions (NC)	R1–R4
	Phosphorylation-dephosphorylation (PdP)	R5–R8
	Stressful Conditions (SC)	R9–R11
Module 2: Secondary Mechanism (M2: SM)	Normal Conditions (NC)	R15–R17
	Phosphorylation-dephosphorylation (PdP)	R18–R21
	Stressful Conditions (SC)	R22–R24
Module 3: GDP-GTP exchange (M3)		R12–R14

Under stressful conditions, we added the PdP reactions to PM and SM. We showed by the CRNT analysis that only PM with PdP reactions can exhibit bistable dynamics, and we attributed this to translation recovery and attenuation (not termination). The PdP reactions of SM and PM and the GDP-GTP/Met-tRNA modules exhibited tristable dynamics, which we attributed to translation recovery and attenuation as a two-step process. We mapped the PM and SM in the tristable dynamics. We found that the occurrence of tristable dynamics is due to a complex interplay of GDP-GTP exchange, $\frac{kin_{T}}{PP_{T}}$, and importantly, $\frac{e5_{T}}{e2B_{T}}$ ratios. Though CRNT analysis indicated the presence of positive feedback and provided kinetic constants that give rise to bistability, it cannot unravel the presence of tristability. However, we mapped the presence of tristability directly to the molecular controls acting on primary and secondary fail-safe mechanisms.

Estimating kinetic parameters and identifying whether they exhibit robust bistable dynamics is difficult since our model is huge and has many unknown kinetic parameters. Existing methods, suitable for identifying bistable dynamics for two or 3-dimensional systems, are not amenable to analyzing our models. Therefore, we estimated the kinetic parameters by taking the steady state and time series data. We estimated 10 parameters from the steady state data of ISR and 6 parameters from the time series, and the rest of the parameters were adjusted during the fitting of time series data. From these kinetic parameter values, the model exhibited robust tristable dynamics. Therefore, most of our model parameters are within the experimental range.

We do not have any concrete evidence from the literature to corroborate our bistability results in the translation initiation and attenuation process. This remains only a hypothesis. However, we are confident that the network is capable of exhibiting bistability because the network we used in our work to perform our analysis was constructed from the experiments, and the data for simulation are obtained by fitting the data to the model. Through our modelling, we provided insight into network bistability and tristability and explained the biological importance of multistability in the translation initiation process. Importantly, the network has only implicit positive feedback loops; therefore, it is not straightforward to determine the presence or absence of bistability by visual inspection of the network. Therefore, our model explanation remains a hypothesis until the experiment validation confirms it.

Both PM and SM, under normal conditions, did not exhibit bistability, as evidenced by CRNT analysis. However, we obtain bistability when GDP/GTP/tRNA reactions couple them, and tristability seems absent. The presence of positive feedback is a necessary but not sufficient condition for a network to exhibit bistability. The implicit positive feedback in our model is due to the push-pull effect of PM and SM coupled with GDP/GTP reactions. But one main difference is that our normal mechanistic model is not a PdP reaction, but a push-pull effect of PM and SM happens due to a complex interaction of e2GDP and TC with e5 and e2B. This sequestration reaction happens in both PM and SM, and the push-pull effect occurs when coupled with GDP/GTP reactions, creating implicit positive feedback. So, this is a different mechanism that generates implicit positive feedback that differs from other similar mechanisms [24,27] In the case of ISR, the scenario is different. This is a PdP reaction where, unlike in the normal case, both PM and SM exhibit bistability and together exhibit tristability. We believe that in the case of PM and SM, the mechanism is akin to a double negative feedback loop similar to the Markevich et al. model of dual phosphorylation-dephosphorylation reactions [28]. When the two bistable systems from PM and SM in ISR are coupled, tristability is obtained. This was first shown by Swat in his thesis [29]. However, Barik et al. [30] have shown the existence of a tristable system by taking simple network motifs that provide insight into conditions for tristable occurrence. We believe that in our model, the tristability is due to the fusion of two implicit positive feedback motifs, namely PM and SM PdP reactions, similar to the Markevich et al model.

In summary, the contribution of this work is that we built a realistic signalling network of only the translation initiation process of both normal and ISR based on extensive experimental data and showed the presence of implicit positive feedback. The network is complex, and therefore, we modularised the network into three parts so that the analyses helped to identify the role of each module, namely PM, SM, and GDP-GTP exchange reactions. Further, we showed the importance of bistability and tristability since translation is an important process that requires regulation.

## Supporting information

S1 Fig1A.NET The .NET CRNT file for the network in Fig 1A. (NET)

S2 Fig1D.NET The .NET CRNT file for the network in Fig 1D. (NET)

S3 Fig2A.NET The .NET CRNT file for the network in Fig 2A. (NET)

S4 Fig3A.NET The .NET CRNT file for the network in Fig 3A. (NET)

S5 Fig3D.NET The .NET CRNT file for the network in Fig 3D. (NET)

S6 Fig4.NET The .NET CRNT file for the network in Fig 4. (NET)

S7 Fig1A.ode The XPPAUT ode files for the network in Fig 1A. (ODE)

S8 Fig1D.ode The XPPAUT ode files for the network in Fig 1D. (ODE)

S9 Fig2A.ode The XPPAUT ode files for the network in Fig 2A. (ODE)

S10 Fig3A.ode The XPPAUT ode files for the network in Fig 3A. (ODE)

S11 Fig3D.ode The XPPAUT ode files for the network in Fig 3D. (ODE)

S12 Fig4.ode The XPPAUT ode files for the network in Fig 4. (ODE)

S13 ISR.cps The COPASI file for the network in Fig 4. (CPS)

S14 Supplementary.pdf The supplementary pdf. (PDF)

Fit_data.xlsx Fit_data.xlsx (XLSX)

Parameters_table.xlsx Parameters_table.xlsx (XLSX)
