# Supplementary material for: Kinetic modelling reveals the presence of multistability in normal and stressful conditions in translational initiation mechanism
Source: PLoS One. 2025 Mar 21;20(3):e0319280. doi: 10.1371/journal.pone.0319280 (PMC11927921; doi:10.1371/journal.pone.0319280)
Supplement: S14 Supplementary.pdf — (PDF) [file pone.0319280.s014.pdf]

Supplementary: Kinetic modelling reveals the presence of multi-stability in normal and stressful conditions in translational initiation mechanism

Range of kinetic parameters for tristability

In the tristability, with the increase in total kinase, the translation attenuation takes place in two jumps, and with the decrease in the total kinase, translation recovery takes place in one jump. This is possible when the kinase concentration at SN1 is less than SN3 and SN2 is greater than SN4. We were interested to look at the range of the individual kinetic parameters that would give rise this tristability. We got the range for each parameter by plotting the two parameter bifurcation between every kinetic parameter and the total kinase.

For example, the Fig S1 shows the two parameter bifurcation between kb4 and kin_T. The region between the green lines represents the bistability caused by the primary mechanism (PM), bounded by SN1 and SN2. The region between the red lines represents the bistability caused by the secondary mechanism (SM), bounded by SN3 and SN4. The region between the two dashed black line, from kb4 = 604.4 $nM^{-1}s^{-1}$ to kb4 = 850.5 $nM^{-1}s^{-1}$, the kinase concentration at SN1 (right green line) is less than SN3 (right red line) and SN2 (left green line) is greater than SN4 (left red line). We have iterated the same method for all the 44 kinetic parameters to get the range of each kinetic parameter that gives rise to tristability where attenuation takes place in two jumps and the recovery takes place in one jump. The ranges of the parameters are given in Table-2 of the main text.

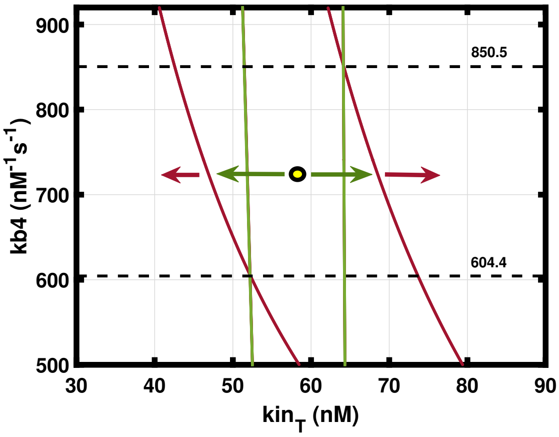

Figure S1: Range for tristability, Two parameter bifurcation between the kinetic parameter kb4 and total kinase (kin_T). The region within green lines represents the bistability by the primary mechanism, bounded by SN1 on the right and SN2 on the left. The region within red lines represents the bistability by the secondary mechanism, bounded by SN3 on the right and SN4 on the left. The yellow dot represents the neutral check point. To the right of the neutral check point, translation attenuates and to the left, translation recovers. The dashed lines represents the lower and upper bound of kb4 which gives tristability where kinase concentration at SN1 < SN3, and SN2 > SN4.

Parameter estimation from the steady state and time series data

Our model has 24 reactions and 44 parameters. There are overall 26 ODEs and 8 conservation relationship. We extracted most of the model parameters from the available experimental steady state and time series data experiments. We extracted the parameters by fitting the steady state and time series data. We used Copasi to fit the experimental data [1].

For fitting both steady state and time series data we used Copasi's inbuilt function "weighted sum of squares" to minimize the error E of the parameters P, which is given by the equation below.

$$E(P) = \sum_{i,j} w_j \cdot (x_{i,j} - y_{i.j}(P))^2$$

In the above equation, $x_{i,j}$ is the point from the dataset, and $y_{i,j}(P)$ is the simulated value with i and j denoting the rows and columns of the dataset. The weight w_j is determined by the inbuilt function weight "Mean Square" given in the below equation.

$$w_j = \frac{1}{< x_j^2 >}$$

We used Evolutionary Programming method in Copasi to carry out the parameter optimization, and used MATLAB (MathWorks, Natick, MA, USA) to generate the error bands.

Steady state data

We took the steady state data from the experiments for the reactions R3, R10, R14, R16, and R23 to estimate the kinetic constants [2, 3]. For this, we first extracted the data points using the ImageJ software [4]. We used Copasi to fit the extracted steady state data of the above reactions. The results of the steady state fits are given in Fig S2.

Time series data

We fit the time-series data got from the experiments for the variables ternary complex, eIF2, and eIF2α. We have taken the full model, and we did not change the 10 parameters of the reactions already fitted from the steady state data, and the rest of the parameters were estimated using Copasi by fixing the upper and lower bounds as the range of the kinetic parameters that gives rise to tristability. For that, we first converted the units of concentration into nM and time to secs to fit the experimental time series data. We took the time series of the ternary complex from [5] (Figure-1, curve eIF2+eIF2B). We converted the % of Met-tRNA bound in the y-axis to nM concentration. In the experiments, 100% represents 240nM (1.2 pmol per 5 µl) in concentration ($y = 240 \times y_p$ where, y is the calculated Y data point in nano molar and y_p is the given Y data point in percentage).

We took the eIF2 time series from [6] (figure 5, curve D15(200µg)), and the y-axis was given in percentage (100% represents 15nM). We converted the % of y-axis of both the control and experiment to the concentration units in the following way.

The calculated Y data point in nano molar for eIF2 is given by the equation below.

$$y = \left(\frac{15 \times y_p}{100}\right) - \left(15 - \frac{y_c \times 15}{100}\right) \tag{1}$$

where,

y is the calculated Y data point in nano molar

y_p is the given Y data point in percentage of the curve D15(200µg)

y_c is the given Y data point in percentage of the control curve

The time series for $eIF2_p$ was taken from figure 2C of [7]. The y-axis is already given in nano molar. The results of the time series fits are given in Fig S3

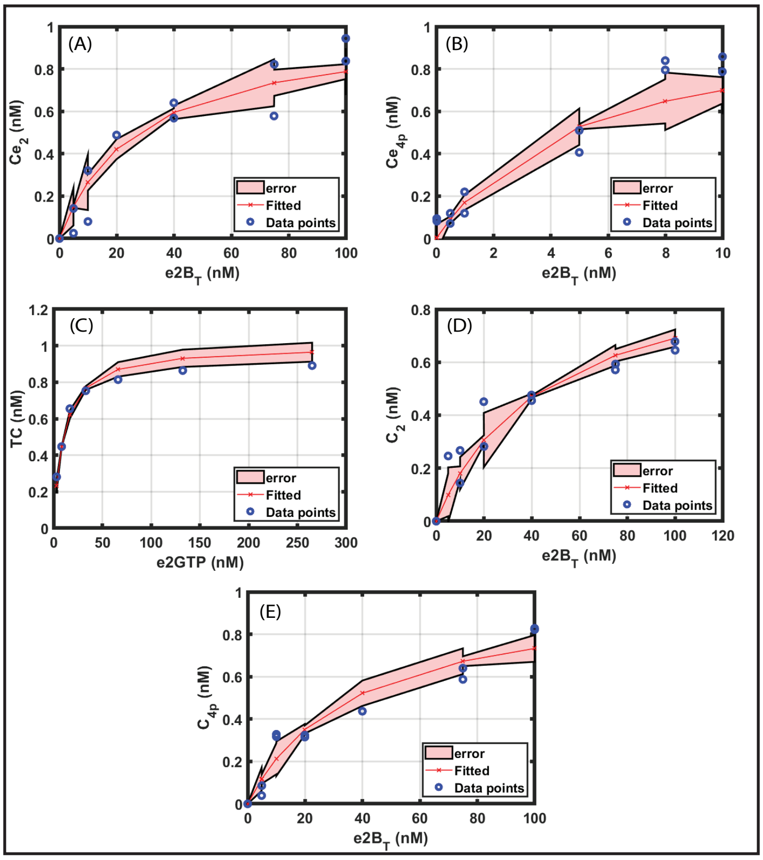

Figure S2: Parameter estimation using Steady state experimental data. The fits of the steady state data using the reactions from the model. **(A)** R_3 was used to fit the steady state of the complex Ce_2 with increasing concentrations of $e2B_T$ [2]. **(B)** With increasing concentrations of $e2B_T$, the steady state of Ce_{4p} was fit using the reaction R_{10} [2]. **(C)** Reaction R_{14} was used to fit the steady state of ternary complex with increasing concentrations of e2GTP [3]. **(D)** R_{16} was used to fit the steady state of the complex C_2 with increasing concentrations of $e2B_T$ [2]. **(E)** Steady state of C_{4p} was fit using reaction R_{23} with increasing concentrations of $e2B_T$ [2].

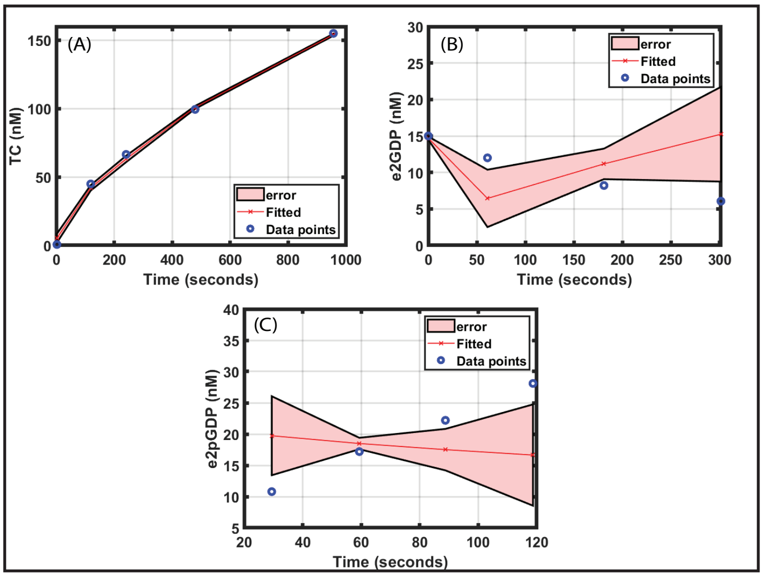

Figure S3: Time series data used to fit in parameters. **(a)** Fit of the data obtained from the time series of ternary complex [5]. **(b)** Fit of the data obtained from the time series of eIF2[3^H]GDP [6]. **(c)** Fit of the data obtained from the time series of the dephosphorylation of eIF2(αP) [7].

References

[1] Stefan Hoops et al. "COPASI—a COmplex PAthway SImulator". *Bioinformatics* 22.24 (2006), pp. 3067–3074. DOI: 10.1093/bioinformatics/btl485.

[2] Martin D Jennings et al. "Fail-safe control of translation initiation by dissociation of eIF2α phosphorylated ternary complexes". *eLife* (2017). DOI: 10.7554/eLife.24542.

[3] Lee D. Kapp and Jon R. Lorsch. "GTP-dependent Recognition of the Methionine Moiety on Initiator tRNA by Translation Factor eIF2". *J. Mol. Biol.* (2004). DOI: 10.1016/j.jmb.2003.11.025.

[4] Curtis T. Rueden et al. "ImageJ2: ImageJ for the next generation of scientific image data". *BMC Bioinformatics* 18.1 (2017), p. 529. DOI: 10.1186/s12859-017-1934-z.

[5] F Les Erickson et al. "Minimum Requirements for the Function of Eukaryotic Translation Initiation Factor 2". *Genetics* (2001). DOI: 10.1093/genetics/158.1.123.

[6] A Alcázar et al. "Changes in the phosphorylation of eukaryotic initiation factor 2 alpha, initiation factor 2B activity and translational rates in primary neuronal cultures under different physiological growing conditions". *Brain Res. Mol. Brain Res.* (1996). DOI: 10.1016/0169-328x(95)00335-p.

[7] N. V. Jammi. "Phosphorylation of the RNA-dependent protein kinase regulates its RNA-binding activity". *Nucleic Acids Res.* (2001). DOI: 10.1093/nar/29.14.3020.
